# Effect of wheat species (*Triticum aestivum* vs *T. spelta*), farming system (organic vs conventional) and flour type (wholegrain vs white) on composition of wheat flour – Results of a retail survey in the UK and Germany – 2. Antioxidant activity, and phenolic and mineral content

**DOI:** 10.1016/j.fochx.2020.100091

**Published:** 2020-05-04

**Authors:** Juan Wang, Eleni Chatzidimitriou, Liza Wood, Gultakin Hasanalieva, Emilia Markelou, Per Ole Iversen, Chris Seal, Marcin Baranski, Vanessa Vigar, Laura Ernst, Adam Willson, Manisha Thapa, Bronwyn J. Barkla, Carlo Leifert, Leonidas Rempelos

**Affiliations:** a Human Nutrition Research Centre, Human Nutrition Research Centre, Institute of Cellular Medicine, Newcastle upon Tyne NE2 4HH, UK; b School of Agriculture and Biology, Shanghai Jiao Tong University, China; c School of Agriculture, Food & Rural Development, Nafferton Ecological Farming Group, Newcastle University, Nafferton Farm, Stocksfield, UK; d French Agency For Food Environmental And Occupational Health and Safety, France (ANSES), Regulated Products Assessment Department, Residues And Food Safety Unit, France; e Department of Sustainable Crop and Food Protection, Faculty of Agriculture, Food and Environmental Sciences, Universita Catollica del Sacro Cuore, I-29122 Piacenza, Italy; f Benaki Phytopathological Institute (BPI), Athens, Greece; g Department of Nutrition, Institute of Basic Medical Sciences, University of Oslo, Oslo, Norway; h Department of Haematology, Oslo University Hospital, Oslo, Norway; i Department of Functional and Organic Food, Institute of Human Nutrition Sciences, Warsaw University of Life Sciences, Nowoursynowska 159c, 02-776 Warsaw, Poland; j Centre for Organics Research, Southern Cross University, Military Rd., Lismore, NSW, Australia; k Southern Cross Plant Science, Southern Cross University, Military Rd., Lismore, NSW, Australia

**Keywords:** Wheat flour, Whole-grain, Organic, Antioxidants, Phenolics, Minerals

## Abstract

There is strong evidence for health benefits from whole-grain wheat consumption and these have been linked to their higher fibre, antioxidant/(poly)phenolic and mineral contents. However, there is still scientific controversy about the relative effect of wheat species (*Triticum aestivum* vs *T. spelta*) and production methods (conventional vs organic) on the nutritional composition of wheat. The retail survey reported here showed that, the use of spelt varieties and organic production results in significantly higher (between 10 and 64%) levels of phenolics and some minerals in wheat flour. However, the relative effect of removing the outer bran and germ during milling was substantially larger; levels of antioxidant activity, and many phenolic compounds and mineral nutrients were 2–5 times higher in whole-grain than white flour. Organic flour contained higher concentrations of the undesirable metals Al and Ni (12% and 81% respectively), and spelt flour had 28% higher concentrations of the toxic metal Cd.

## Highlights

• Whole-grain flours had 2–5 times higher antioxidant and mineral levels than white flour.
• Organic flour had higher antioxidant and mineral levels than conventional flour.
• Organic flour had a higher Al and Ni content than conventional flour.
• Spelt wheat had higher antioxidant and mineral levels than common wheat flour.
• Spelt wheat had a 28% higher Cd content than conventional flour.

## 1 Introduction

Cereal products account for approximately 45% of the total daily calorie intake of humans globally, ranging from around 25% in many European countries (e.g. in Germany and the UK) to around 55% in some developing countries (e.g. India) (National Geographic, 2019). In Europe, common wheat (*Triticum aestivum* L.) is the most important cereal species used for human consumption. Wheat flour is the main ingredient in many staple food products such as breakfast cereals, pancakes, pasta, noodles, bread and other bakery products. Most of these foods are made from refined grains, where the outer grain layers (the bran and germ) are removed from the endosperm before further processing (e.g. milling to produce “white” flour) (Jones and Engleson, 2010, National Geographic, 2019).

However, the use of cereal products made from whole-grain flour (where the whole grain is milled) is increasingly recommended by nutritionists, because there is now substantial evidence for higher whole-grain consumption resulting in a reduced risk of obesity, type 2 diabetes, certain cancers and cardiovascular diseases (Cho et al., 2013, Jones and Engleson, 2010).

The health benefits from whole-grain consumption are thought to be mainly associated with the higher fibre, mineral (e.g. Zn, Cu and Se) and (poly)phenol/antioxidant content (which is mainly in the bran fraction of the grain) of whole-grain flour (Jones & Engleson, 2010).

A recent meta-analyses of published data found evidence that organic production methods result in significantly higher antioxidant/(poly)phenolic and Zn, but lower protein, nitrate, nitrite and Cd concentrations in cereals (Baranski et al., 2014, Cooper et al., 2011, Rempelos et al., 2018). In addition older, longer straw, spring wheat varieties (those used before the 1960s) which are preferred by some organic farmers, were reported to produce grain with significantly higher concentrations of mineral micro-nutrients (Zn, Cu and Se) than modern, short-straw varieties (Murphy, Reeves, & Jones, 2008).

Spelt wheat (*Triticum spelta* L.) is a husked-wheat species which in the past was widely grown in Northern Europe but is now considered a minor cereal. Spelt wheat has recently increased its production area and market share in Europe, especially in organic farming (Escarnot, Jacquemin, Agneessens, & Paquot, 2012). This is thought to be mainly due to (a) its ability to grow under low input conditions (which make it particularly suitable for organic farming systems) and (b) consumer perceptions that spelt wheat has a higher nutritional value compared with common wheat (Dean et al., 2007).

There have been several studies in which the macronutrient and mineral composition, fibre, lipid, protein, phenolic compounds and yield of common and/or spelt wheat grain, flour or food products made from them were analysed. However, there is large within-species variation in both *T. aestivum* and *T. spelta*, and some *T. spelta* varieties have originated from crosses with *T. aestivum*. As a result there is still considerable uncertainty about whether or not and to what extent spelt wheat has a superior nutritional composition compared with common wheat (Calzuola et al., 2013, Escarnot et al., 2012, Wang, 2019).

To our knowledge, there are no comparative retail surveys in which (a) the composition of common and spelt wheat-based food samples that were collected in the same retail outlets are compared and (b) confounding factors such as wheat farming system (e.g. organic vs conventional), grain processing/flour type (e.g. white or whole-grain) were considered in the survey design.

The **overall aim** of the study was to obtain a more accurate estimate of the difference in phytochemical and mineral concentrations between spelt and common wheat grain by analysing all accessible retail brands of wheat flour available in Germany and the UK (thereby estimating differences between the common and spelt wheat varieties currently used), while accounting for the confounding effects of farming system and flour type.

The **specific objectives** of this study were therefore to (a) compare antioxidant activity, and protein, (poly)phenolic and mineral concentrations in white and whole-grain common and spelt wheat flour brands/products available in the UK and Germany (b) to study the effect of primary production methods (organic vs conventional) on antioxidant activity, and protein (poly)phenolic and mineral micronutrient concentrations in wheat flour and (c) identify potential interactions between wheat species, primary production protocols, and post-harvest processing/milling with respect to antioxidant activity, and protein, (poly)phenolic, and mineral concentrations.

The minerals assessed included all plant macro- and micronutrients, the undesirable elements aluminium and nickel and the toxic metal cadmium.

The study tested 4 main hypotheses, which were: (a) whole-grain wheat flour has higher antioxidant activity, and protein, phenolic and mineral nutrient concentrations than white flour, (b) organic wheat flour has higher antioxidant activity, and phenolic and mineral nutrient concentrations, but lower protein, Cd and Ni concentrations than conventional wheat flour with the relative differences between organic and conventional brands being greater for whole-grain than white flour and (c) spelt wheat flour has higher antioxidant activity, and protein, phenolic and mineral nutrient concentrations than common wheat flour and (d) the relative impact of processing on antioxidant/phenolic and mineral nutrient concentrations is substantially greater that the impact of farming system and wheat species choice.

## 2 Materials and methods

### 2.1 Retail survey design

The retail survey of wheat flour was conducted over two successive years in 2015 and 2016, but no common wheat samples were collected in Germany in 2015. As a result, differences between countries could only be assessed in 2016. In total, 168 samples were purchased from supermarkets in the UK (Tesco, Waitrose, Sainsbury, Marks&Spencer, Holland& Barrett, Fenwick Food Hall) and Germany (Budnikowsky, Demeter, Denn’s Biomarket, Dm, Edeka, Kaufland, LIDL, Netto, Rewe, REAL 2015, Vitalia 2015) and websites in the UK (Allinson, Amazon, Bacheldre Watermill, Buywholefoodonline, Gilchester online, Matthews Gotswold, Sharpham Park, Shipton Mill online, Wessex Mill) in the same period in each year (see Supplementary Material Table S1 for the number of flour samples/brands analysed for each flour type in the UK and Germany).

The experimental design included three factors/variables: wheat species (*T. aestivum* or *T. spelta*), farming system (organic or conventional) and flour type (white or whole-grain). Cereal brands were used as replicates, with only one sample per brand (supermarket own or manufacturers brands) being used for each combination of wheat species, farming system, flour type per year. This was primarily done to avoid pseudo-replication, since the use of more than one sample per brand could have resulted in both flour samples originating from the same batch of grain used by the millers; different brands were assumed to have been made by different mills or at least from different grain batches. Samples were transferred from original packages to vacuum food bags and then stored in a −80 °C freezer in containers with silica gel prior to analysis.

### 2.2 Nitrogen and protein content analysis

Grain N concentrations were determined by the total combustion method using a vario MACRO cube C/N Analyzer (Elementar LTD, Germany). Around 50 mg of homogenised fresh sample was weighed into a tin foil cup. The cup was carefully folded and squashed into a pellet to expel the air using a tool provided by Elementar. Before each run, a set of control standards were run to ensure that the analyser was working correctly. Results obtained for grain *N*-content were multiplied by 6.25 to obtain grain protein concentration as recommended by Simonne, Simonne, Eitenmiller, Mills, and Cresman (1997).

### 2.3 Phenolic/antioxidant analysis

#### 2.3.1 Extraction of phenolic acid fractions

Three separate phenolic acid fractions (soluble free, soluble conjugated, and insoluble bound) were extracted from the flour samples using the method described by Li, Shewry, and Ward (2008). All extractions for each sample were repeated in triplicate. A detailed description of the extraction protocols for each fraction are provided in the supplementary information.

#### 2.3.2 Total phenolics/phenolic acid content

The total phenolics content in wheat grain was quantified using the Folin-Ciocalteu method (Singleton, Orthofer, & Lamuela-Raventós, 1999) with minor modifications. Standard solutions of gallic acid were prepared as follows: 20 g of gallic acid were dissolved in 3 mL methanol then made up to volume with distilled water in a 100 mL flask. The standards were serially diluted for generation of a standard calibration curve. The concentration of standards for serial dilutions were 200 µg/mL, 100 µg/mL, 50 µg/mL, 25 µg/mL, 12.5 µg/mL, 6.25 µg/mL and 3.125 µg/ml.

Twenty µL of each sample solution, the serial standard solutions and distilled water as blank were added to wells on a 96-well microplate. Each standard solution and sample solution was tested in duplicate. To each well 130 µL of Folin-Ciocalteu reagent (diluted by distilled water 1:10 (v/v)), was then added, followed after 5 min by 100 µL of 7.5% sodium carbonate (Na_2_CO_3_) solution. The microplates were then covered with a plastic cover and incubated in the dark at 40 °C for 30 min. The absorbance of all solutions was measured at 760 nm with a spectrophotometric microplate reader (Konica Minolta, Tokyo). Final results were presented as µmol gallic acid equivalent (GAE) per g flour (DW).

#### 2.3.3 Total flavonoid content

The total flavonoid content of wheat extracts was determined by a colorimetric method described previously (Liu et al., 2002) with minor modification. Catechin (15 g) was dissolved in a 100 mL flask with 10 mL methanol then made up to volume with distilled water. The standards were serially diluted to create a standard calibration curve. The concentration of standard from serial dilutions were 150 µg/mL, 75 µg/mL, 37.5 µg/mL, 18.75 µg/mL, 9.375 µg/mL, 4.6875 µg/mL and 2.34375 µg/mL.

Twenty five µL of each sample solution, the serial standard solutions and distilled water as blank were loaded on a 96-well microplate. Each standard solution and sample solution was run in duplicated. 7.5 µL of a 5% NaNO_2_ solution was added to all sample solutions, standards and blanks. After 6 min at room temperature, 15 µL of 10% AlCl_3_·6H_2_O solution was added and the mixture was allowed to stand for another 5 min at room temperature. Then 50 µL of 1 M NaOH were added to the mixture followed by 152.5 µL distilled water. The absorbance was measured immediately at λ 510 nm using the spectrophotometric microplate reader (Konica Minolta). The results were expressed as µmol catechin equivalent (CE) per g flour (DW).

#### 2.3.4 Total antioxidant activity by TEAC and FRAP

Total antioxidant activity of wheat extracts was measured by both the TEAC and FRAP methods (Benzie and Strain, 1996, Re et al., 1999). Detailed descriptions of the TEAC and FRAP protocols are provided in the supplementary information.

### 2.4 Phenolics profile analysis by HPLC

Extraction for phenolics profile analysis by high-performance liquid chromatography (HPLC) was as described in the phenolic/antioxidant analysis section previously. All chemicals were purchased from Sigma-Aldrich. Phenolic acid standards (protocatechuic acid, 4-hydroxybenzoic acid, vanillic acid, syringic acid, p-coumatic acid, syringaldehyde, sinapic acid and ferulic acid) were prepared as a stock solution at 0.1 mg/mL in 70:30 methanol:water, and were stored at −20 °C in the dark until HPLC analysis was performed, within three months of extraction.

Flour extracts were analysed by HPLC on a Shimadzu Prominence HPLC system equipped with an LC-20AD pump, SIL-20AC antosampler, and SPD-M20A photodiode array detector (Shimadzu Crop., Kyoto, Japan). Data collection and integration were performed using LabSolution software. Phenolic acids were separated on a reverse-phase Thermo Scientific Hypersil C18 column (250 × 4.6 mm, 5 µm particle size). The column was heated at 25 °C while the samples tray temperature was set to 4 °C.

Mobile phase A was acetonitrile, while mobile phase B was 0.1% acetic acid. The gradient programme for the mobile phase (A:B) was at 0.02 min (5:95), 10 min (20:80), 15 min (25:75), 20 min (35: 65), 25 min (65: 36), 25.01 (100:0), 30 min (100:0), 30.01 (5:95) and 40 (5:95). The flow rate of the mobile phase was 2 mL/m, and the injection volume was 20 µL. Scanning was performed from 190 nm to 800 nm, and phenolic acids were identified by comparing retention times and UV–VIS spectra with those of pure standards. Concentrations, expressed in µg/g DW, were calculated at 230, 270 or 320 nm using calibration curves of phenolic acid standards. The following phenolic acid peaks were identified and quantified according to their spectra and relative retention time: 4-hydroxyvalproic acid, vanillic acid, syringic acid, p-coumaric acid, syringaldeyde, sinapic acid, ferulic acid. The sum of all phenolic acids detected by HPLC was used as an estimate for the total phenolic acid concentration in wheat grain.

### 2.5 Analysis of macro and micro mineral nutrients

#### 2.5.1 Digestion

Flour (0.25 g) was mixed with 5 mL 69% nitric acid (HNO_3_) in teflon vessels then digested in a microwave reaction unit (CEM-Mars 6, USA) in “vegetable” mode with a four step heating program (step 1 ramp to 180 °C; step 2 hold 180 °C for 10 min; step 3 ramp to 205 °C for 20 min; step 4 cooling down). After digestion, samples were allowed to cool to room temperature and then were filtered through blue ribbon quantitative filter paper (Whatman Grade 589/3), the filtrates were mixed with distilled water and diluted with ultrapure water in 50 mL flasks. Digested solutions were stored in Sterilin tubes at 4 °C until analysis.

#### 2.5.2 Analysis by ICP

Macro and micro minerals in digested solutions were analysed with an Inductively Coupled argon Plasma Optical Emission Spectrometer (ICP-OES) equipped with a CCD detector (Vista-Pro Axial; Varian Pty Ltd., Mulgrave, Australia). Analytical quality was checked against the certified values of the quality reference material (wheat flour SRM 1567a and apple leaves SRM1515) which were included in every batch of 40 samples.

### 2.6 Statistical analysis

Analysis of variance (ANOVA) derived from Linear mixed-effects (lme) models (Pinheiro & Bates, 2000) was used to assess the effects and interactions between factors on measured parameters by using the *‘nlme’* package in R (R Core Team, 2018). Samples were collected over two years (2016 and 2017) but white spelt flour was not available in Germany during 2016. Since it was not possible to include all experimental factors in a single analysis, three separate 3-factor ANOVAs were carried out. (a) ANOVA 1 with wheat species (common vs spelt wheat), farming system (organic vs conventional) and flour type (whole-grain vs white) as factors; (b) ANOVA 2 with country (UK vs Germany), wheat species (common vs spelt wheat) and farming system (organic vs conventional) as factors for the UK whole-grain wheat samples only (to estimate potential confounding effects of country) and (c) ANOVA 3 with year (2015 vs 2016), wheat species (common vs spelt wheat) and farming system (organic vs conventional) as factors for the 2016 whole-grain wheat samples only (to estimate potential confounding effects of year/production season). The hierarchical nature of the experimental design was designated in the random error structures of the model as: replicate (commercial brand)/year, country/wheat species/farming system (ANOVA 1); replicate (brand)/year/wheat species (ANOVA 2) and replicate (brand)/country/wheat species (ANOVA 3). For all parameters it was also checked that the residuals were normally distributed by using the *‘qqnorm’* function in R.

In order to further investigate the significant (*p* < 0.05) interactions between factors, general linear hypothesis tests (Tukey contrasts) were performed using the *‘glht’* function of the *‘multcomp’* package (Bretz, Hothorn, & Westfall, 2011) in R. The experimental design was reflected in the same random error structures used for the lme models. This method allows multiple comparisons in unbalanced models with arbitrary error distribution and hence arbitrary data distribution and variance structure.

## 3 Results

### 3.1 Phytochemical concentrations and antioxidant activity

Significant main effects of wheat species were only detected for total phenolic content (colorimetric assay) and antioxidant activity (TEAC), with spelt having an 11% higher phenolic content and 15% higher antioxidant activity (TEAC) (Table 1). Significant main effects of farming system and flour type were detected for concentrations of all phytochemical groups, ferulic and sinapic acid (the dominant phenolic compounds found in wheat grain) and total antioxidant activity (FRAP and TEAC) (Table 1). Phenolic/antioxidant concentrations and activity were found to be between 10 and 33% higher in organic compared with conventional flour and between 2 and 4.3 times higher in whole-grain compared with white flour (Table 1). Similar trends were detected when (a) free, bound and conjugated fractions were compared and (b) other individual phenolic compounds (including protocatechuic acid, 4-hydroxybenzoic acid, vanillic acid, syringic acid, p-coumatic acid, sinapic acid) and syringaldehyde were quantified by HPLC (Tables S3–S20 and S24–S29).

**Table 1:** Main effect means ± SE and *p*-values for the effects and interaction between wheat species, farming system and flour type on concentrations of phenolic compounds and total antioxidant activity (FRAP, TEAC) in wheat flour (results are expressed on a flour dry weight basis).

Factor	Colorimetric Assays		HPLC			Colorimetric Assays	
	Total phenolic content	Flavonoids	Ferulic acid	Sinapic acid	total phenolic compounds*	Antioxidant activity	
						FRAP	TEAC
	µmol GAE g^−1^ flour	µmol catechin g^−1^ flour	µmol g^−1^ flour	µmol g^−1^ flour	µmol g^−1^ flour	µmol FeSO_4_ 7H_2_O g^−1^ flour	µmol Trolox g^−1^ flour
**Species**							
Spelt (n = 55)	9.4 ± 0.4	0.98 ± 0.08	369 ± 34	28 ± 2.6	438 ± 39	5.1 ± 0.4	9.8 ± 0.8
Wheat (n = 112)	8.5 ± 0.4	0.95 ± 0.08	352 ± 30	29 ± 2.4	419 ± 35	4.8 ± 0.4	8.5 ± 0.6

**Farming system**							
Conventional (n = 84)	8.4 ± 0.4	0.83 ± 0.08	334 ± 34	26 ± 2.6	394 ± 40	4.3 ± 0.4	7.8 ± 0.6
Organic (n = 83)	9.2 ± 0.4	1.10 ± 0.09	382 ± 31	31 ± 2.5	457 ± 36	5.5 ± 0.4	10.1 ± 0.7

**Flour Type**							
White (n = 90)	6.0 ± 0.2	0.57 ± 0.07	120 ± 8	12 ± 1.2	149 ± 10	2.0 ± 0.1	3.9 ± 0.2
Whole-grain (n = 77)	12.1 ± 0.3	1.42 ± 0.07	636 ± 23	48 ± 1.9	749 ± 26	8.3 ± 0.2	14.8 ± 0.4

**ANOVA-results (p-values)**							
*Main Effects*							
Species (SP)	0.0053	NS	NS	NS	NS	NS	0.038
Farming System (FS)	0.0232	0.0094	NS	0.0299	NS	<0.0001	<0.0001
Flour Type (FT)	<0.0001	<0.0001	<0.0001	<0.0001	<0.0001	<0.0001	<0.0001

*Interactions*							
SP × FS	NS	NS	NS	NS	NS	NS	NS
SP × FT	<0.0001^1^	NS	0.0002^1^	<0.0001^1^	0.0001	0.0002^1^	0.0112^1^
FS × FT	NS	NS	NS	NS	NS	0.0131^2^	NS
SP × FS × FT	NS	0.0198^3^	0.0062^3^	NS	0.0109^3^	NS	NS

Total phenolic acids by HPLC is the sum concentration of protocatechuic acid, 4-hydroxybenzoic acid, vanillic acid, syringic acid, p-coumaric acid, syringaldehyde, sinapic acid and ferulic acid detected by HPLC.

1 See Table 2 for interaction means ± SE.

2 see Table 3 for interaction means ± SE.

3 See Table 4 for interaction means ± SE.

Significant 2-way interactions between wheat species and flour type were detected for concentrations of all phytochemicals (except flavonoids) and antioxidant activity (FRAP and TEAC) (Table 1). When whole-grain flour was compared, common wheat flour had significantly higher phytochemical concentrations and antioxidant activity than spelt flour. In contrast, when white flour was compared spelt flour had numerically higher phytochemical concentrations and antioxidant activity, but the difference was only significant for total phenolics (Table 2).

**Table 2:** Interactions means ± SE for the effects of species and flour type on total phenolic, ferulic acid and sinapic acid concentrations, and antioxidant activity (FRAP and TEAC) in flour collected from UK and DE in 2015 and 2016. (results are expressed on a flour dry weight basis).

Parameters assessed	Factor 1	Factor 2	
	Wheat species	Flour type	
		White	Whole-grain
Total phenolic (Colorimetric) µmol GAE g^−1^ flour (DW)	Spelt	7.1 ± 0.4 B a	11.2 ± 0.4 A b
	Common	5.6 ± 0.2 B b	12.7 ± 0.4 A a
Total ferulic acid (HPLC) µmol g^−1^ flour (DW)	Spelt	131 ± 24 B a	554 ± 26 A b
	Common	116 ± 7 B a	691 ± 32 A a
Total sinapic acid (HPLC) µmol g^−1^ flour (DW)	Spelt	14 ± 3 B a	39 ± 2 A b
	Common	11 ± 1 B a	54 ± 4 A a
Antioxidant activity FRAP µmol FeSO_4_ 7H_2_O g^−1^ flour (DW)	Spelt	2.2 ± 0.3 B a	7.4 ± 0.3 A b
	Common	1.9 ± 0.1 B a	8.9 ± 0.3 A a
Antioxidant activity TEAC µmol Trolox g^−1^ flour (DW)	Spelt	4.5 ± 0.5 B a	13.8 ± 0.6 A b
	Common	3.7 ± 0.2 B a	15.4 ± 0.6 A a
Sodium* mg kg^−1^	Spelt	22 ± 3 B a	54 ± 3 A b
	Common	20 ± 1 B a	65 ± 2 A a
Calcium mg g^−1^	Spelt	0.33 ± 0.07 A b	0.27 ± 0.01 A a
	Common	0.66 ± 0.06 A a	0.31 ± 0.02 B a
Sulphur mg g^−1^	Spelt	1.04 ± 0.05 A a	0.95 ± 0.05 A a
	Common	0.72 ± 0.03 B b	0.84 ± 0.04 A a
Molybdenum mg kg^−1^	Spelt	0.38 ± 0.03 A a	0.44 ± 0.05 A a
	Common	0.27 ± 0.02 B b	0.43 ± 0.03 A a

*Excludes data of four self-rising flour samples.

For each parameter assessed means labelled with the same capital letter within rows and lower-case letter within columns are not significant different (Turkey’s honestly significant difference test P < 0.05).

A significant 2-way interaction between farming system and flour type was only detected for antioxidant activity (FRAP) (Table 1). Antioxidant activity was significantly higher in organic than conventional whole-grain but not white flour (Table 3).

**Table 3:** Interactions means ± SE for the effects of flour type and farming system on total antioxidant activity (FRAP) in flour collected from UK and DE in 2015 and 2016 (results are expressed on a flour dry weight basis).

Parameters assessed	Factor 1	Factor 2	
	Farming system	Flour type	
		White	Whole-grain
Antioxidant activity FRAP (µmol FeSO_4_ 7H_2_O g^−1^ flour)	Organic	2.0 ± 0.2 B a	8.7 ± 0.3 A a
	Conventional	2.0 ± 0.2 B a	7.8 ± 0.3 A b
Phosphorus mg g^−1^	Organic	0.95 ± 0.06 B a	2.37 ± 0.12 A a
	Conventional	0.83 ± 0.07 B a	1.85 ± 0.14 A b
Potassium mg g^−1^	Organic	1.0 ± 0.1 B a	2.3 ± 0.1 A a
	Conventional	0.9 ± 0.1 B a	2.0 ± 0.1 A b
Magnesium mg g^−1^	Organic	0.24 ± 0.02 B a	0.76 ± 0.04 A a
	Conventional	0.21 ± 0.02 B a	0.58 ± 0.04 A b
Manganese mg kg^−1^	Organic	7 ± 0.6 B a	22 ± 1.3 A a
	Conventional	6 ± 0.6 B a	16 ± 1.2 A b
Zinc mg kg^−1^	Organic	9 ± 0.7 B a	22 ± 1.0 A a
	Conventional	8 ± 0.6 B a	15 ± 1.1 A b
Copper mg kg^−1^	Organic	4.1 ± 0.3 B a	6.3 ± 0.4 A a
	Conventional	3.3 ± 0.3 B a	4.4 ± 0.3 A b
Aluminium mg kg^−1^	Organic	4.2 ± 0.4 A a	4.9 ± 0.5 A a
	Conventional	4.2 ± 0.3 A a	3.0 ± 0.3 A b

For each parameter assessed means labelled with the same capital letter within rows and lower-case letter within columns are not significant different (Turkey’s honestly significant difference test P < 0.05).

Significant 3-way interactions were detected for total flavonoids, ferulic acid concentrations and total phenolic compounds concentration detected by HPLC (Table 1). Significantly higher flavonoid concentration in organic compared with conventional samples were only detected for white common wheat flour. In contrast, significantly higher ferulic acid concentrations in conventional compared with organic samples were only detected for whole-grain common wheat flour. For all other flour types there was no significant differences between organic and conventional samples (Table 4). Significantly higher concentrations of ferulic acid in common wheat compared with spelt wheat flour were only detected in conventional whole-grain flour, while significantly higher concentrations of total phenolic compounds in common wheat compared to spelt wheat flour were detected in both organic and conventional whole-grain flour samples (Table 4).

**Table 4:** Interactions means ± SE for the effects of species, farming system and flour type on the total flavonoid, ferulic content and total concentration of phenolic acids detected by HPLC of flour collected from UK and DE between 2015 and 2016 (results are expressed on a flour dry weight basis).

Parameter assessed	Factor 1	Factor 2	Factor 3	
	Species	Flour type	Farming system	
			Organic	Conventional
*Colorimetric Assays*				
Total flavonoids µmol catechin g^−1^ flour (DW)	Spelt	White	0.46 ± 0.07 A b	0.60 ± 0.09 A b
		Whole-grain	1.49 ± 0.11 A a	1.05 ± 0.11 A a
	Wheat	White	0.78 ± 0.20 A b	0.45 ± 0.05 B b
		Whole-grain	1.45 ± 0.08 A a	1.51 ± 0.21 A a

*HPLC*				
Total Ferulic acid µmol g^−1^ flour (DW)	Spelt	White	102 ± 16 A b	159 ± 45 A c
		Whole-grain	579 ± 25 A a	509 ± 58 A b
	Wheat	White	123 ± 12 A b	112 ± 9 A c
		Whole-grain	650 ± 36 B a	735 ± 52 A a

Total phenolic compounds µmol g^−1^ flour (DW)	Spelt	White	132 ± 20 A c	197 ± 56 A c
		Whole-grain	677 ± 29 A b	603 ± 66 A b
	Wheat	White	153 ± 15 A c	136 ± 12 A c
		Whole-grain	779 ± 41 A a	856 ± 58 A a

For each parameter assessed means labelled with the same capital letter within rows and lower case letter within columns are not significant different (Turkey’s honestly significant difference test P < 0.05).

When concentrations of free, bound and conjugated phenolic, flavonoid, concentrations and antioxidant activity in these fractions were compared, overall trends were broadly similar to those found for total concentrations and activity (Tables S3–S20 and S24–S29).

### 3.2 Protein and mineral macro-nutrient concentrations

#### 3.2.1 Protein

Protein concentrations were estimated based on nitrogen (N) concentrations in grains and ANOVA results were therefore identical for protein and *N*-content (Table 5). Significant main effects were detected for all 3 factors and protein/N concentrations were significantly higher in spelt, conventional and whole-grain flour (Table 5).

**Table 5:** Main effect means ± SE and *p*-values for the effects and interaction between year, cereals species, farming system and bran type on protein and mineral macronutrient concentrations in wheat flour collected in the UK and DE in 2015 and 2016 (results are expressed on a flour dry weight basis).

Factor	Protein	mineral macro-nutrients						
		N	Na*	P*	K	S	Ca*	Mg
	%	mg g^−1^	mg g^−1^	mg g^−1^	mg g^−1^	mg g^−1^	mg g^−1^	mg g^−1^
**Species**								
Spelt (n = 55)	11.5 ± 0.2	18.2 ± 0.3	29.5 ± 1.4	1.9 ± 0.1	1.77 ± 0.11	0.99 ± 0.04	0.29 ± 0.03	0.54 ± 0.04
Wheat (n = 110)	10.7 ± 0.2	16.9 ± 0.3	43.1 ± 2.8	1.2 ± 0.1	1.39 ± 0.08	0.77 ± 0.02	0.52 ± 0.04	0.38 ± 0.03

**Farming system**								
Conventional (n = 83)	11.3 ± 0.2	17.9 ± 0.3	40.2 ± 2.9	1.2 ± 0.1	1.26 ± 0.08	0.82 ± 0.03	0.46 ± 0.04	0.35 ± 0.03
Organic (n = 82)	10.5 ± 0.2	16.7 ± 0.3	36.7 ± 2.6	1.7 ± 0.1	1.78 ± 0.10	0.86 ± 0.03	0.43 ± 0.04	0.52 ± 0.04

**Flour Type**								
White (n = 89)	10.5 ± 0.2	16.6 ± 0.3	37.0 ± 2.4	0.9 ± 0.1	0.90 ± 0.04	0.80 ± 0.03	0.58 ± 0.05	0.22 ± 0.01
Whole-grain (n = 76)	11.5 ± 0.2	18.2 ± 0.3	40.2 ± 3.2	2.2 ± 0.1	2.23 ± 0.08	0.88 ± 0.03	0.29 ± 0.01	0.68 ± 0.03

**ANOVA (*p*-values)**								
*Main Effects*								
Species (SP)	0.0003	0.0003	0.0023	<0.0001	0.0004	<0.0001	0.0004	<0.0001
Farming System (FS)	0.0001	0.0001	NS	<0.0001	0.0001	NS	NS	<0.0001
Flour Type (FT)	<0.0001	<0.0001	0.0228	<0.0001	<0.0001	<0.0001	<0.0001	<0.0001

*Interactions*								
SP × FS	NS	NS	NS	NS	NS	0.0014^3^	NS	NS
SP × FT	NS	NS	0.0366^1^	NS	NS	0.0036^1^	0.0029^1^	NS
FS × FT	NS	NS	NS	0.0470^2^	0.0339^2^	NS	NS	0.0155^2^
SP × FS × FT	NS	NS	NS	NS	NS	NS	NS	NS

*Excludes data of four self-risicommonmmmng flour samples; NS, not significant.

1 See Table 2 for interaction means ± SE.

2 See Table 3 for interaction means ± SE.

3 see Table S2 for interaction means ± SE.

##### 3.2.1.1 Sodium (Na)

Significant main effects of wheat species and flour type were detected for Na concentrations, which were significantly higher in common wheat than spelt flour, and whole-grain than white flour (Table 5).

There was a 2-way interaction between wheat species and flour type. Significantly higher sodium concentration in common wheat than spelt wheat were only detected in whole-grain flour but not in white flour samples (Table 2).

##### 3.2.1.2 Phosphorus (P), potassium (K), magnesium (Mg), sulphur (S)

Significant main effects of all 3 experimental factors were detected for P, K and Mg concentrations, which were significantly higher in spelt compared with common wheat flour (58, 27, and 42% respectively), organic compared with conventional flour (42, 27, and 49% respectively) and whole-grain compared with white flour (144, 125, and 209% respectively) (Table 5). Similar trends were also detected for S, but main effects were only significant for wheat species and flour type; S concentrations were also significantly higher in spelt (29%) and whole-grain flour (10%) (Table 5).

For P, K and Mg, there were significant 2-way interactions between farming system and flour type (Table 5), with significantly higher P, K and Mg concentrations in organic compared with conventional flour being detected in whole-grain, but not white flour samples (Table 3).

For S there were significant 2-way interactions between (a) wheat species and flour type and (b) wheat species and farming system (Table 5). Significantly higher S-concentrations in wholegrain than white flour were detected in common, but not spelt wheat samples (Table 2). Also, S-concentrations were higher in conventional than organic spelt wheat flour, but higher organic than conventional common wheat flour (Table S2).

##### 3.2.1.3 Calcium (Ca)

For Ca a significant interaction between wheat species and flour type was detected (Table 5). White common wheat flour had significantly (~2-time) higher Ca concentrations than white spelt, and wholegrain common and spelt wheat flour (Table 2).

### 3.3 Mineral micro-nutrients

Significant main effects of all 3 experimental factors were detected for manganese (Mn), zinc (Zn), copper (Cu) and Molybdenum (Mo) concentrations, which were significantly higher in spelt compared with common wheat flour (31, 64, 35 and 24% respectively), organic compared with conventional flour (51, 45, 43 and 70% respectively) and whole-grain compared with white flour (216, 111, 49 and 43% respectively) (Table 6). Similar trends were also detected for Iron (Fe), but main effects were only significant for farming system and flour type; Fe concentrations were also significantly higher in organic compared with conventional flour (16%) and whole-grain compared with white flour (63%) (Table 6).

**Table 6:** Main effect means ± SE and *p*-values for the effects and interaction between wheat species, farming system and flour type on concentrations of mineral micronutrients and undesirable/toxic metals in wheat flour collected in the UK and Germany in 2015 and 2016 (results are expressed on a flour dry weight basis).

	Mineral micronutrients					Undesirable and toxic metals		
	Fe	Mn	Zn	Cu	Mo	Al	Ni	Cd
	mg kg^−1^	mg kg^−1^	mg kg^−1^	mg kg^−1^	mg kg^−1^	mg kg^−1^	mg kg^−1^	μg/kg
**Species**								
Spelt (n = 55)	22 ± 1	14.7 ± 1.3	18 ± 1.2	5.4 ± 0.4	0.41 ± 0.03	3.8 ± 0.3	0.46 ± 0.05	46 ± 3
Wheat (n = 110)	20 ± 1	11.2 ± 0.9	11 ± 0.6	4.0 ± 0.2	0.33 ± 0.02	4.3 ± 0.3	0.32 ± 0.06	36 ± 2

**Farming system**								
Conventional (n = 83)	19 ± 1	9.9 ± 0.8	11 ± 0.7	3.7 ± 0.2	0.27 ± 0.01	3.7 ± 0.2	0.26 ± 0.02	38 ± 2
Organic (n = 82)	22 ± 1	14.9 ± 1.1	16 ± 1.0	5.3 ± 0.3	0.46 ± 0.03	4.6 ± 0.3	0.47 ± 0.08	42 ± 2

**Flour Type**								
White (n = 89)	16 ± 1	6.2 ± 0.4	9 ± 0.5	3.7 ± 0.2	0.30 ± 0.02	4.2 ± 0.2	0.36 ± 0.07	38 ± 2
Whole-grain (n = 76)	26 ± 1	19.6 ± 1.0	19 ± 0.9	5.5 ± 0.3	0.43 ± 0.03	4.1 ± 0.3	0.36 ± 0.04	42 ± 2

**ANOVA- results (p-values)**								
*Main Effects*								
Species (SP)	NS	0.0015	<0.0001	0.0024	0.0096	NS	NS	0.0041
Farming System (FS)	0.0123	<0.0001	<0.0001	<0.0001	<0.0001	0.0094	0.0216	NS
Flour Type (FT)	<0.0001	<0.0001	<0.0001	<0.0001	<0.0001	NS	NS	NS

*Interactions*								
SP × FS	NS	NS	NS	0.0242^3^	NS	0.0109^3^	NS	NS
SP × FT	NS	NS	NS	NS	0.0465^1^	NS	NS	NS
FS × FT	NS	0.0024^2^	0.0006^2^	0.0324^2^	NS	0.0015^2^	NS	NS
SP × FS × FT	NS	NS	NS	NS	NS	NS	NS	NS

NS, not significant.

1 See Table 2 for interaction means ± SE.

2 See Table 3 for interaction means ± SE.

3 See Table S2 for interaction means ± SE.

For Mo a significant 2-way interaction between wheat species and flour type was detected (Table 6), with concentration found to be significantly higher in spelt than common white wheat flour, but not whole-grain flour (Table 2).

For Mn, Zn and Cu significant 2-way interactions were detected between farming systems and flour type (Table 6), with concentration found to be significantly higher in organic than conventional whole-grain, but not white flour (Table 3).

For Cu a significant 2-way interaction was also detected between wheat species and farming systems (Table 6). Cu concentration were significantly higher in organic than conventional common wheat, but not spelt wheat flour (Table S2).

### 3.4 Undesirable (Al, Ni) and toxic (Cd) metals

A significant main effect of wheat species (but not farming system and flour type) was detected for the toxic metal cadmium (Cd), with concentrations found to be significantly higher (28%) in spelt than common wheat flour (Table 6). Significant main effects of farming system (but not wheat species and flour type) were detected for the undesirable metals Al and Ni, with concentrations found to be significantly higher (12 and 81% respectively) in organic compared to conventional flour (Table 6).

For Al there were significant 2-way interactions between (a) farming system and flour type and wheat species and farming system (Table 6). Al concentrations were found to be significantly higher in organic than conventional whole-grain but not white four samples (Table 6). Al concentration were significantly higher in organic than conventional common wheat, but not spelt wheat flour (Table S2). Also, common wheat had higher Al concentrations than spelt wheat flour when organic, but not when conventional samples were compared (Table S2).

## 4 Discussion

In the wheat flour survey reported here, all accessible brands of white and wholegrain flour available in the UK and Germany were analysed. Results therefore reflect the (a) variability associated with the currently used spelt and common wheat varieties, agronomic practices and milling protocols used by commercial farmers and processors in these countries and (b) range of wheat flour quality available to consumers. However, since information on location of farms, crop varieties and specific agronomic practices used by organic and conventional farmers and post-harvest drying, storage, cleaning, and milling protocols used for organic and conventional grains were not recorded, it was not possible to assess to what extent these parameters affected the flour composition parameters measured.

Overall, results from this study suggest that there are significant differences in antioxidant and mineral composition between (a) spelt and common wheat and (b) organic and conventional flour and that refining of grains (removal of most of the bran and germ) during the production of white flour removes a large proportion of these nutrients. The relative impacts and interactions between wheat genetics, agronomic practices and grain processing on the nutritional quality and associated potential health impacts are discussed in separate sections below.

### 4.1 Effect of grain refining/processing (white vs whole-grain flour)

The results of this study which showed that whole-grain flour has a substantially higher nutritional value than white flour is consistent with previous studies, which reported that whole-grain flour and cereal products have a substantially higher protein, fibre, phenolic and mineral macro-, and micronutrient content than white flour and cereal products (Cho et al., 2013, Jones and Engleson, 2010, Oghbaei and Prakash, 2016). Results also suggest that refining (removal of the bran and germ) (a) has a substantially greater impact on the mineral nutrients and phytochemical concentrations in flour than wheat genetics/species choice (*T. aestivum* vs *T. spelta*) and production protocols (organic vs conventional), (b) diminishes the differences in antioxidant activity, and phenolic and mineral concentrations in wheat flour produced with grain from contrasting farming systems and wheat species.

Similar to several previous studies (Brier et al., 2015, Brunetti et al., 2012) the survey reported here also showed that refining does not increase concentrations of nutritionally undesirable and/or toxic metals (Al, Ni, Cd) in flour, which may indicate that these compounds are more evenly distributed between the endosperm, germ and bran fraction of cereal grains as suggested by Brier et al. (2015). In contrast, a recent study by (Ertl & Goessler, 2018) reported (a) higher Al, Cd and Ni concentrations in wholemeal compared to white spelt flour, (b) higher Ni concentrations in wholemeal than white common wheat flour, and (c) no significant effect of refining on Al and Cd concentrations in common wheat flour, and Al, Cd and Ni concentrations in rye flour. However, the reasons for the inconsistent results found in different studies and/or for contrasting wheat species are currently unknown.

The finding that phenolic, ferrulic acid, sinapic acid and antioxidant activity (FRAP and TEAC) were higher in common than spelt whole-grain samples, but higher in spelt than common wheat white flour is reported for the first time here. It suggests that the negative impact of grain refining on antioxidant levels is greater in common than spelt wheat. This view is supported by the finding that the difference in N/protein concentrations between whole-grain and white flour was also greater in common (11%) than spelt (3%) wheat. This could be due to differences in (a) grain physiology/morphology and/or (b) the amount of bran and germ being removed in the refining process used for spelt and common wheat grain as suggested in previous reports (Longin et al., 2016).

It is well known that changes to the refining process affect the percentage of bran and germ loss and thus nutritional quality and it has been recommended that studies focused on comparing the nutritional composition of white flour use standardised extraction and analytical methods (Shewry & Hey, 2015) to allow more accurate comparisons between wheat species/varieties and/or farming systems.

### 4.2 Effect of wheat species (common vs spelt wheat)

Results from this study suggest that spelt flour had slightly, but significantly higher phenolic concentrations and antioxidant activity than common wheat, and that the differences were greater in whole-grain than white flour. Concentrations of protein/N, S, most mineral micro-nutrients analysed and cd were also significantly higher in spelt than common wheat. The results reported here are consistent with some but not all previous studies that compared the composition of spelt and common wheat flour. For example, Abdel-Aal and Rabalski (2008) found higher antioxidant and total phenolic concentrations and Calzuola et al. (2013) reported significantly higher flavonoid concentrations in spelt compared to common wheat, while other studies reported no significant differences in antioxidant/phytochemical levels between species (Li et al., 2008, Shewry and Hey, 2015).

Most previous comparative studies used milled whole-grain or whole-grain wheat products made from a range of different *T. aestivum* and *T. spelta* cultivars/genotypes and it is likely that results were affected by within species variation, which can be considerable (Abdel-Aal & Rabalski, 2008).

Also, many previous studies only assessed or reported concentrations of antioxidant activity, total phenolic, flavonoid and phenolic acid concentration in free fractions (Van Hung, 2014), while in the study reported here, free, bound and conjugated fractions were quantified. This may also explain differences in outcomes between studies.

The finding of substantially higher Ca concentrations in white (but not wholemeal) common compared to spelt wheat flour, may indicate that only white common wheat flour is fortified with Ca in the UK and Germany.

### 4.3 Effect of farming systems (organic versus conventional)

The finding of higher poly(phenolic), Mg and Zn concentrations, and/or antioxidant activity in organic compared with conventional wheat flour, is consistent with the results of a *meta*-analysis of data from 343 peer-reviewed publications, which reported that antioxidant/(poly-phenolic) concentrations were higher in organic than conventional crops (Baranski et al., 2014). However, the *meta*-analysis and a recent factorial field experiment (Cooper et al., 2011) also suggested that concentrations of Ni and the toxic metal Cd are significantly lower in organic cereals crops, while organic flour had similar Cd and higher concentrations of Al and Ni compared with conventional wheat flour in the study reported here. The higher Al and Ni concentrations represent an undesirable trade-off for the higher antioxidant levels in organic flour, but is unlikely to have nutritional/health impact given the relatively low concentrations of Al and Ni found in flour and the level of difference observed (Trumbo, Yates, Schlicker, & Poos, 2001).

There is evidence that use of mineral *N*-fertiliser, herbicides and modern short-straw varieties can all have a negative effect on antioxidant/(poly)-phenolic concentrations in wheat. Mineral *N*-fertiliser use was reported as a major driver for the lower concentrations of (poly)phenolics/antioxidants found in both grains and leaves of conventional cereal crops (Baranski et al., 2014, Rempelos et al., 2018). Also, higher concentrations of phenolic acids and flavonoids in leaves of organic crops were linked to higher levels of resistance against biotrophic cereal diseases such as mildew (Rempelos et al., 2018).

The use of synthetic chemical pesticides in conventional farming may also, at least partially, explain the lower antioxidant/phenolic concentrations in conventional wheat flour reported here. For example, Daniel, Meier, Schlatter, and Frischknecht (1999) reported that herbicides reduce concentrations of phenolic compounds and other secondary metabolites in plants.

Longer straw US common wheat varieties developed in the 1960 were shown to have higher mineral micronutrient concentrations than modern short straw varieties currently used in the US (Murphy et al., 2008). The use of contrasting wheat varieties in organic and conventional farming systems could also be a possible explanation for the composition differences between organic and conventional flour.

However, since detailed information on the agronomic practices and wheat varieties used to make the flour assessed in this study was not available, the relative effect of genetic/variety choice and specific agronomic (e.g. fertilisation and crop protection regimes) drivers on the wheat flour composition detected in this study could not be determined.

### 4.4 Potential impacts on human health

The most recent review of the role of phenolics/antioxidants in modern nutrition by Williamson (2017) stated the absorption and metabolism of phenolics/antioxidants in body and summarised biological effects of phenolics/antioxidants-rich tea, coffee and cocoa indicated by human intervention studies. The gut microbiota plays a critical role in absorption of many phenolics/antioxidants and more than 80% of a dose can be absorbed and ultimately excreted in the urine (Williamson, 2017).

There is now substantial epidemiological evidence that a diet high in phenolics/antioxidants-rich food protects against developing cardiovascular disease and type 2 diabetes (de Munter et al., 2007, Williamson, 2017). However, since many of the antioxidant rich foods contain a great diversity of biologically active phytochemicals it is sometimes difficult to separate out the effects of individual compounds (Williamson, 2017). Also, despite extensive research, the exact mechanisms of action of phenolics/antioxidants in the human body is not completely understood, but there is strong evidence that some targets such as nitric oxide metabolism, carbohydrate digestion and oxidative enzymes are important for the health benefits observed (Williamson, 2017).

Cereals and cereal-based foods are important sources of minerals in the diet (Albergamo et al., 2018, Oghbaei and Prakash, 2016). However, the processing of grains has a major impact on the resulting mineral content, depending on the degree of extraction of flours (Ertl and Goessler, 2018, Oghbaei and Prakash, 2016). In some countries loss of some minerals is addressed through mandatory fortification of refined flours with some but not all of minerals lost during processing. Since the majority of phytochemicals are contained in bran and germ fractions, these are also lost during the refining process (Oghbaei & Prakash, 2016). Thus, as expected, in the present study mineral concentrations were significantly higher in whole-grain compared with white flours. Whilst there is strong evidence for the health benefits of consuming whole grains in reducing the risk of chronic disease, there is limited (or no) evidence on the effects of whole-grain consumption on mineral status in humans. Higher intakes of micronutrient minerals is encouraged for most populations where intake often falls short of national recommendations (Albergamo et al., 2018, Ertl and Goessler, 2018). The results of this study suggest that higher mineral intakes could be achieved by switching from (a) white to whole-grain, (b) conventional to organic and (c) common to spelt flours. Although not determined in the current study, whole-grain flours have a higher fibre content than white flours underlining the benefit of consuming whole-grain flours in order to increase fibre intake which globally is below recommendations (Cho et al., 2013).

The higher phenolic and antioxidant activity in organic wheat flour may, at least partially explain the results of recent cohort studies which compared health outcomes in individuals with low and high levels of organic food consumption. These studies reported significant positive associations between high levels of organic food consumption and lower risks of obesity, metabolic syndrome, pre-eclampsia and eczema, hypospadias and cancer (Baranski et al., 2017, Baudry et al., 2018a, Baudry et al., 2018b). However, results from these cohort studies also indicated that organic consumers have better diets/show closer compliance with dietary recommendations such as increasing fruit and vegetable and whole-grain consumption. This suggests that both higher whole-grain and organic food consumption may explain the associations observed. This view is supported by the finding that conventional wholemeal wheat flour contains substantially higher pesticide residues than conventional white flour, while very low levels of pesticide residues are found in both white and whole-grain organic wheat flour in the UK and Germany (Wang, 2019). Only consumption of organic whole-grain flour therefore allows higher intakes of antioxidant/(poly)phenolics and mineral micro-nutrients without simultaneously increasing dietary exposure to pesticides.

## 5 Conclusion

The finding of substantially higher antioxidant concentrations and essential mineral micronutrients such as Mg, Fe, Zn, Cu in whole-grain flour supports hypothesis 1 and lends further support to current dietary recommendations to switch to whole-grain cereal product consumption.

The finding of higher antioxidant activity and/or phenolic and mineral nutrient concentrations in organic compared with conventional, and spelt compared with common wheat flour supports hypotheses 2 and 3. Since the flour survey reported here was based on all spelt and common wheat flour brands that could be found on the shelves of major UK and German retailers, the results can be assumed to accurately reflect the nutritional composition of different wheat flour products available in these countries. The study therefore also suggests that organic and spelt flour consumption would result in higher antioxidant and mineral micronutrient intakes and associated potential health benefits.

The finding of similar Cd and higher Ni concentrations in organic compared with conventional flour does not support hypothesis 2 and contradicts the findings of a recent systematic review/*meta*-analysis which reported that Cd and/or Ni concentrations are higher in conventional cereal (see Introduction).

The finding that grain processing (removal of the bran and germ during milling to produce white flour) has a substantially larger impact on nutritionally desirable antioxidant activity, and phenolic and mineral nutrient concentrations than primary production protocols and wheat species choice supports hypothesis 4.

## CRediT authorship contribution statement

**Juan Wang:** Conceptualization, Data curation, Formal analysis, Investigation, Methodology, Visualization, Writing - original draft, Writing - review & editing. **Eleni Chatzidimitriou:** Investigation, Methodology, Supervision. **Liza Wood:** Investigation. **Gultakin Hasanalieva:** Investigation, Methodology. **Emilia Markelou:** Conceptualization, Writing - review & editing. **Per Ole Iversen:** Validation, Writing - review & editing. **Chris Seal:** Methodology, Project administration, Resources, Supervision, Validation, Writing - review & editing. **Marcin Baranski:** Data curation, Formal analysis, Validation. **Vanessa Vigar:** Conceptualization, Writing - review & editing. **Laura Ernst:** Writing - review & editing. **Adam Willson:** Writing - review & editing. **Manisha Thapa:** Writing - review & editing. **Bronwyn J. Barkla:** Validation, Writing - review & editing. **Carlo Leifert:** Conceptualization, Funding acquisition, Project administration, Resources, Supervision, Validation, Writing - original draft, Writing - review & editing. **Leonidas Rempelos:** Data curation, Formal analysis, Investigation, Supervision, Visualization, Writing - original draft, Writing - review & editing.

## Declaration of Competing Interest

The authors declare that they have no known competing financial interests or personal relationships that could have appeared to influence the work reported in this paper.
