# Supplementary material for: Effect of wheat species (Triticum aestivum vs T. spelta), farming system (organic vs conventional) and flour type (wholegrain vs white) on composition of wheat flour – Results of a retail survey in the UK and Germany – 2. Antioxidant activity, and phenolic and mineral content
Source: Food Chem X. 2020 May 4;6:100091. doi: 10.1016/j.fochx.2020.100091 (PMC7215096; doi:10.1016/j.fochx.2020.100091)
Supplement: Supplementary data 1 [file mmc1.docx]

**SUPPLEMENTARY INFORMATION**

Contents

[**Methods** 1](#_Toc22568871)

[***Phenol/antioxidant extraction protocols*** 6](#_Toc22568872)

[***Antioxidant activity (TEAC and FRAP) analysis protocols*** 6](#_Toc22568873)

[**Results** 8](#_Toc22568874)

[***ANOVA 1: wheat species × farming system × flour type*** 10](#_Toc22568875)

[***ANOVA 2: country × wheat species × farming system (for whole-grain flour only)*** 38](#_Toc22568876)

[***ANOVA 3: year (2015, 2016) × wheat species × farming system (for UK whole-grain flour only)*** 49](#_Toc22568877)

# Table List

[***Table S1*.**  Number (n) of wheat flour brands sampled and analysed for antioxidant activity and phenolic concentrations (A&P) and mineral content (MIN) in the retail survey in the UK and Germany in 2015 and 2016. 6](#_Toc36644543)

[***Table S2*.** Interactions means ± SE for the effects of wheat species and farming system on copper concentrations in flour collected from UK and DE in 2015 and 2016 (results are expressed on a flour dry weight basis). 7](#_Toc36644544)

[***Table S3*.** Main effect means ± SE and *p*-values for the effects and interaction between wheat species, farming system and flour type on phenolic content of flour collected from UK and DE between 2015 and 2016 (results are expressed on a flour dry weight basis) 8](#_Toc36644545)

[***Table S3.1*.** Interactions means ± SE for the effects of wheat species and flour type on phenolic content in flour collected from UK and DE between 2015 and 2016 (results are expressed on a flour dry weight basis) 12](#_Toc36646734)

[***Table S4*.** Main effect means ± SE and *p*-values for the effects and interaction between wheat species, farming system and flour type on Antioxidant activity by FRAP of flour collected from UK and DE between 2015 and 2016 (results are expressed on a flour dry weight basis) 10](#_Toc36644546)

[***Table S4.1.*** Interactions means ± SE for the effects of wheat species and flour type on antioxidant activity by FRAP of flour collected from UK and DE between 2015 and 2016 (results are expressed on a flour dry weight basis) 11](#_Toc36645644)

[***Table S4.2.*** Interactions means ± SE for the effects of farming system and flour type on antioxidant activity by FRAP of flour collected from UK and DE between 2015 and 2016 (results are expressed on a flour dry weight basis) 11](#_Toc36645645)

[***Table S5*.** Main effect means ± SE and *p*-values for the effects and interaction between wheat species, farming system and flour type on Antioxidant activity by TEAC of flour collected from UK and DE between 2015 and 2016 (results are expressed on a flour dry weight basis) 12](#_Toc36644547)

[***Table S5.1.*** Interactions means ± SE for the effects of wheat species and flour type on antioxidant activity by TEAC of flour collected from UK and DE between 2015 and 2016 (results are expressed on a flour dry weight basis) 13](#_Toc36645649)

[***Table S6.*** Main effect means ± SE and *p*-values for the effects and interaction between wheat species, farming system and flour type on flavonoid content in flour collected from UK and DE between 2015 and 2016 (results are expressed on a flour dry weight basis) 14](#_Toc36644548)

[***Table S6.1.*** Interactions means ± SE for the effects of farming system and flour type on antioxidant activity by flavonoid in flour collected from UK and DE between 2015 and 2016 (results are expressed on a flour dry weight basis) 15](#_Toc36645658)

[***Table S6.2.*** Interactions means ± SE for the effects of wheat species, farming system and flour type on flavonoid content in bound fraction of flour collected from UK and DE between 2015 and 2016 (results are expressed on a flour dry weight basis) 15](#_Toc36645659)

[***Table S7.*** Main effect means ± SE and *p*-values for the effects and interaction between wheat species, farming system and flour type on ferulic acid detected by HPLC in flour collected from UK and DE between 2015 and 2016 (results are expressed on a flour dry weight basis) 16](#_Toc36644549)

[***Table S7.1.*** Interactions means ± SE for the effects of wheat species and flour type on ferulic phenolic acid concentration detected by HPLC in flour collected from UK and DE between 2015 and 2016 (results are expressed on a flour dry weight basis) 18](#_Toc36645664)

[***Table S7.2.*** Interactions means ± SE for the effects of farming system and flour type on ferulic acid concentration detected by HPLC in flour collected from UK and DE between 2015 and 2016 (results are expressed on a flour dry weight basis) 18](#_Toc36645665)

[***Table S7.3*.** Interactions means ± SE for the effects of wheat species, flour type and farming system on ferulic phenolic acid concentration detected by HPLC in flour collected from UK and DE between 2015 and 2016 (results are expressed on a flour dry weight basis) 19](#_Toc36645666)

[***Table S8.*** Main effect means ± SE and *p*-values for the effects and interaction between wheat species, farming system and flour type on Sinapic acids detected by HPLC in flour collected from UK and DE between 2015 and 2016 (results are expressed on a flour dry weight basis) 19](#_Toc36644550)

[***Table S8.1*.** Interactions means ± SE for the effects of wheat species and flour type on sinapic acid concentration detected by HPLC in flour collected from UK and DE between 2015 and 2016 (results are expressed on a flour dry weight basis) 21](#_Toc36645680)

[***Table S8.2*.** Interactions means ± SE for the effects of wheat species, flour type and farming system on sinapic acid concentration in flour collected from UK and DE between 2015 and 2016 (results are expressed on a flour dry weight basis) 21](#_Toc36645681)

[***Table S9*.** Main effect means ± SE and *p*-values for the effects and interaction between wheat species, farming system and flour type on 4-hydroxybenzoic acid concentration detected by HPLC in flour collected from UK and DE between 2015 and 2016 (results are expressed on a flour dry weight basis) 21](#_Toc36644551)

[***Table S9.1.*** Interactions means ± SE for the effects of wheat species and flour type on 4-hydroxybenzoic acid concentration detected by HPLC in flour collected from UK and DE between 2015 and 2016 (results are expressed on a flour dry weight basis) 23](#_Toc36645692)

[***Table S9.2*.** Interactions means ± SE for the effects of farming system and flour type on 4-hydroxybenzoic acid concentration detected by HPLC in flour collected from UK and DE between 2015 and 2016 (results are expressed on a flour dry weight basis) 23](#_Toc36645693)

[***Table S9.3*.** Interactions means ± SE for the effects of wheat species, flour type and farming system on 4-hydroxybenzoic acid concentration detected by HPLC in flour collected from UK and DE between 2015 and 2016 (results are expressed on a flour dry weight basis) 24](#_Toc36645694)

[***Table S10.*** Main effect means ± SE and *p*-values for the effects and interaction between wheat species, farming system and flour type on vanillic acid detected by HPLC in flour collected from UK and DE between 2015 and 2016 (results are expressed on a flour dry weight basis) 24](#_Toc36644552)

[***Table S10.1*.** Interactions means ± SE for the effects of wheat species and flour type on vanillic acid concentration detected by HPLC in flour collected from UK and DE between 2015 and 2016 (results are expressed on a flour dry weight basis) 26](#_Toc36645704)

[***Table S10.2*.** Interactions means ± SE for the effects of wheat species, flour type and farming system on vanillic acid concentration detected by HPLC in flour collected from UK and DE between 2015 and 2016 (results are expressed on a flour dry weight basis) 26](#_Toc36645705)

[***Table S11*.** Main effect means ± SE and *p*-values for the effects and interaction between wheat species, farming system and flour type on syringic acid detected by HPLC in flour collected from UK and DE between 2015 and 2016 (results are expressed on a flour dry weight basis) 26](#_Toc36644553)

[***Table S11.1*.** Interactions means ± SE for the effects of wheat species and flour type on syringic acid concentration detected by HPLC in flour collected from UK and DE between 2015 and 2016 (results are expressed on a flour dry weight basis) 28](#_Toc36645716)

[***Table S11.2.*** Interactions means ± SE for the effects of wheat species, flour type and farming system on syringic acid concentration detected by HPLC in flour collected from UK and DE between 2015 and 2016 (results are expressed on a flour dry weight basis) 28](#_Toc36645717)

[***Table S12*.** Main effect means ± SE and *p*-values for the effects and interaction between wheat species, farming system and flour type on p-coumaric acid concentration detected by HPLC in flour collected from UK and DE between 2015 and 2016 (results are expressed on a flour dry weight basis) 28](#_Toc36644554)

[***Table S12.1*.** Interactions means ± SE for the effects of wheat specie and farming system on p-coumaric acid concentration detected by HPLC in flour collected from UK and DE between 2015 and 2016 (results are expressed on a flour dry weight basis) 30](#_Toc36645729)

[***Table S12.2*.** Interactions means ± SE for the effects of wheat specie and flour type on p-coumaric acid concentration detected by HPLC in flour collected from UK and DE between 2015 and 2016 (results are expressed on a flour dry weight basis) 30](#_Toc36645730)

[***Table S12.3*.** Interactions means ± SE for the effects of farming system and flour type on p-coumaric acid concentration detected by HPLC in flour collected from UK and DE between 2015 and 2016 (results are expressed on a flour dry weight basis) 31](#_Toc36645731)

[***Table S13*.** Main effect means ± SE and *p*-values for the effects and interaction between wheat species, farming system and flour type on syringaldeyde concentration detected by HPLC in flour collected from UK and DE between 2015 and 2016 (results are expressed on a flour dry weight basis) 31](#_Toc36644555)

[***Table S13.1*.** Interactions means ± SE for the effects of wheat species and flour type on syringaldeyde concentration detected by HPLC in flour collected from UK and DE between 2015 and 2016 (results are expressed on a flour dry weight basis) 34](#_Toc36645737)

[***Table S13.2*.** Interactions means ± SE for the effects of wheat species and flour type on syringaldeyde concentration detected by HPLC in flour collected from UK and DE between 2015 and 2016 (results are expressed on a flour dry weight basis) 34](#_Toc36645738)

[***Table S14*.** Main effect means ± SE and *p*-values for the effects and interaction between wheat species, farming system and flour type on total concentration of phenolic acids detected by HPLC in flour collected from UK and DE between 2015 and 2016 (results are expressed on a flour dry weight basis) 33](#_Toc36644556)

[***Table S14.1*.** Interactions means ± SE for the effects of wheat species and flour type on total concentration of phenolic acids detected by HPLC in flour collected from UK and DE between 2015 and 2016 (results are expressed on a flour dry weight basis) 37](#_Toc36646962)

[***Table S14.2*.** Interactions means ± SE for the effects of farming system and flour type on total concentration of phenolic acids detected by HPLC in flour collected from UK and DE between 2015 and 2016 (results are expressed on a flour dry weight basis) 37](#_Toc36646963)

[***Table S14.3*.** Interactions means ± SE for the effects of wheat species, flour type and farming system on total concentration of phenolic acids detected by HPCL in flour collected from UK and DE between 2015 and 2016 (results are expressed on a flour dry weight basis) 38](#_Toc36646964)

[***Table S 15*.**   Number (n) of common and spelt wheat **whole-grain** flour brands sampled and analysed for antioxidant activity, protein, phenolic and mineral micronutrient content in the retail survey in the UK and Germany in 2016. 36](#_Toc36644557)

[***Table S16*.** Main effect means ± SE and *p*-values for the effects and interaction between country (UK and Germany), cereals species and farming system on phenolic content in flour collected in 2016 (results are expressed on a flour dry weight basis) 37](#_Toc36644558)

[***Table S17*.** Main effect means ± SE and *p*-values for the effects and interaction between country (UK and Germany), cereals species and farming system on Antioxidant activity by FRAP in flour collected in 2016 (results are expressed on a flour dry weight basis) 38](#_Toc36644559)

[***Table S18*.** Main effect means ± SE and *p*-values for the effects and interaction between country (UK and Germany), cereals species and farming system on antioxidant activity by TEAC in flour collected in 2016 (results are expressed on a flour dry weight basis) 39](#_Toc36644560)

[***Table S18.1*.** Interactions means ± SE for the effects of country (UK and Germany) and production on total antioxidant activity of flour collected in 2016 by TEAC (results are expressed on a flour dry weight basis) 42](#_Toc36645776)

[***Table S19*.** Main effect means ± SE and *p*-values for the effects and interaction between country (UK and Germany), cereals species and farming system on flavonoid content in flour collected in 2016 (results are expressed on a flour dry weight basis) 41](#_Toc36644561)

[***Table S20*.** Main effect means ± SE and *p*-values for the effects and interaction between country (UK and Germany), cereals species and farming system on Ferulic acid content in flour collected in 2016 (results are expressed on a flour dry weight basis) 42](#_Toc36644562)

[***Table S20.1*.** Interactions means ± SE for the effects of species and farming system on phenolic acid concentration in flour collected in 2016 (results are expressed on a flour dry weight basis) 45](#_Toc36645785)

[***Table S21*.** Main effect means ± SE and *p*-values for the effects and interaction between country (UK and Germany), cereals species and farming system on macro nutrition in wholegrain flour collected in 2016 (results are expressed on a flour dry weight basis) 44](#_Toc36644563)

[***Table S22*.** Main effect means ± SE and *p*-values for the effects and interaction between country (UK and Germany), cereals species and farming system on micro nutrition and toxic metals in wholegrain flour collected in 2016 (results are expressed on a flour dry weight basis) 45](#_Toc36644564)

[***Table S22.1*** Interactions means ± SE for the effects of country (UK and Germany) and species on S and Zn content in flour collected in 2016 (results are expressed on a flour dry weight basis) 48](#_Toc36645793)

[***Table S22.2*** Interactions means ± SE for the effects of country (UK and Germany) and species on Mo content in flour collected in 2016 (results are expressed on a flour dry weight basis) 48](#_Toc36645794)

[***Table S22.3*** Interactions means ± SE for the effects of species and farming systems on Mo and Al content in flour collected from UK and Germany in 2016 (results are expressed on a flour dry weight basis) 48](#_Toc36645795)

[***Table S 23.***  Number (n) of wheat flour brands sampled and analysed for antioxidant activity and phenolic concentration (A&P) and mineral content (MIN) in the retail survey in UK between 2015 and 2016. 47](#_Toc36644565)

[***Table S24.*** Main effect means ± SE and *p*-values for the effects and interaction between year (2015 and 2016), cereals species and farming system on phenolic content in UK whole-grain flour collected between 2015 and 2016 (results are expressed on a flour dry weight basis) 48](#_Toc36644566)

[***Table S25*.** Main effect means ± SE and *p*-values for the effects and interaction year, cereals species and farming system on antioxidant activity by FRAP of UK whole-grain flour collected between 2015 and 2016 (results are expressed on a flour dry weight basis) 49](#_Toc36644567)

[***Table S25.1*.** Interactions means ± SE for the effects of year and farming system on total antioxidant activity by FRAP of UK whole-grain flour collected between 2015 and 2016 (results are expressed on a flour dry weight basis) 52](#_Toc36645801)

[***Table S26*.** Main effect means ± SE and *p*-values for the effects and interaction between year, cereals species and farming system on antioxidant activity by TEAC of UK whole-grain flour collected between 2015 and 2016 (results are expressed on a flour dry weight basis) 51](#_Toc36644568)

[***Table S26.1*.** Interactions means ± SE for the effects of species, farming system and flour type on total antioxidant activity by TEAC of UK whole-grain flour collected between 2015 and 2016 (results are expressed on a flour dry weight basis) 54](#_Toc36645809)

[***Table S27*.** Main effect means ± SE and *p*-values for the effects and interaction between year, cereals species and farming system on flavonoid content in UK whole-grain flour collected between 2015 and 2016 (results are expressed on a flour dry weight basis) 53](#_Toc36644569)

[***Table S28*.** Main effect means ± SE and *p*-values for the effects and interaction between year, cereals species and farming system on Ferulic acid content in UK whole-grain flour collected between 2015 and 2016 (results are expressed on a flour dry weight basis) 54](#_Toc36644570)

[***Table S28.1*.** Interactions means ± SE for the effects of year and species on phenolic acid concentration in UK whole-grain flour collected between 2015 and 2016 (results are expressed on a flour dry weight basis) 57](#_Toc36645816)

[***Table S28.2*.** Interactions means ± SE for the effects of species and flour type on phenolic acid concentration in UK whole-grain flour collected between 2015 and 2016 (results are expressed on a flour dry weight basis) 57](#_Toc36645817)

[***Table S29*.** Main effect means ± SE and *p*-values for the effects and interaction between year, cereals species and farming system on Sinapic acids content in UK whole-grain flour collected between 2015 and 2016 (results are expressed on a flour dry weight basis) 56](#_Toc36644571)

[***Table S29.1*.** Interactions means ± SE for the effects of year, species and farming system on phenolic acid concentration in UK whole-grain flour collected between 2015 and 2016 (results are expressed on a flour dry weight basis) 59](#_Toc36645823)

[***Table S30*.** Main effect means ± SE and *p*-values for the effects and interaction between year, cereals species and farming system on macro nutrient concentrations in UK whole-grain flour collected between 2015 and 2016 (results are expressed on a flour dry weight basis) 58](#_Toc36644572)

[***Table S30.1*.** Interactions means ± SE for the effects of year and species on Mg content in UK whole-grain flour collected between 2015 and 2016 (results are expressed on a flour dry weight basis) 61](#_Toc36646364)

[***Table S30.2*.** Interactions means ± SE for the effects of year and farming system on Na content in UK whole-grain flour collected between 2015 and 2016 (results are expressed on a flour dry weight basis) 61](#_Toc36646365)

[***Table S30.3*.** Interactions means ± SE for the effects of species, farming system and flour type on K and Mg content in UK whole-grain flour collected between 2015 and 2016 (results are expressed on a flour dry weight basis) 61](#_Toc36646366)

[***Table S31*.** Main effect means ± SE and *p*-values for the effects and interaction between year, cereals species and farming system on micronutrient and toxic metal concentrations in UK whole-grain flour collected between 2015 and 2016 (results are expressed on a flour dry weight basis) 60](#_Toc36644573)

[***Table S31.1*.** Interactions means ± SE for the effects of year and species on Zn and Mo content in UK whole-grain flour collected between 2015 and 2016**.** 63](#_Toc22573061)

[***Table S31.2*.** Interactions means ± SE for the effects of year and species on Fe and Mo content in UK whole-grain flour collected between 2015 and 2016**.** 63](#_Toc22573062)

[***Table S31.3*.** Interactions means ± SE for the effects of species and farming systems on Al content in UK whole-grain flour collected between 2015 and 2016 63](#_Toc22573063)

[***Table S31.4*.** Interactions means ± SE for the effects of species, farming system and flour type Zn content in UK whole-grain flour collected between 2015 and 2016. 64](#_Toc22573064)

**Methods**

## ***Phenol/antioxidant extraction protocols***

Soluble Free Fraction: 0.025g of sample was mixed with 1 mL of 80% chilled ethanol for 10 mins by continuous shaking at room temperature followed by sonication in a sonic bath for a further 6 mins. After centrifugation at 13200 rpm for 5 mins, the supernatant was removed and extraction was repeated twice. Supernatants were combined and then evaporated under nitrogen gas flow at 35 ⁰C to dryness and reconstituted in 250 µL of ultrapure water. The extracts were stored at -80⁰C until use.

Bound Fraction: 0.01g of sample was mixed with 1mL of 80% chilled ethanol for 10 mins by continuous shaking at room temperature followed by sonication in a sonic bath for a further 6 mins. After centrifugation at 13200 rpm for 5 mins, the supernatant was removed for conjugate fraction extraction (see below). The remaining residue was then digested with 800 µL 2M sodium hydroxide at room temperature for 4 hours with 1 min shaking each half hour. The mixture was neutralized with 120 µL hydrochloric acid (HCl) and the solution was extracted three times with 800 µL ethyl acetate. The ethyl acetate fraction was evaporated under nitrogen gas flow at 35 ⁰C to dryness. Phenolic compounds were reconstituted in 250 µL water of water and stored at -80 ⁰C until use.

Soluble Conjugated Fraction: ethanol extracts from the bound phenolic extractions process (see above) were used for soluble conjugated extractions. The extracts were dried under nitrogen flow at 35 ⁰C and then were digested with 400 µL 2M NaOH for 4 hours, and the solution was neutralized with 80 µL HCl. The mixture was extracted three times with 500 µL ethyl acetate, and the ethyl acetate fraction was evaporated to dryness at 35 ⁰C under nitrogen gas flow. Phenolics were recovered for analysis in 250 µL water and stored at -80 ⁰C until use.

## ***Antioxidant activity (TEAC and FRAP) analysis protocols***

**TEAC assay.** Trolox (6-hydroxy-2,5,7,8-tet-ramethychroman-2-carboxylic acid) was used as the antioxidant standard. To prepare the standard calibration curve 6.3mg Trolox were dissolved in 50 mL flask using 50% methanol, then were serially diluted by distilled water to concentrations of 126, 63, 31.5, 15.75, 7.875, 3.9375 and 1.96875 µg/mL. **ABTS working solution preparation**: Solution A, 2.45 mM potassium persulfate (K_2_S_2_O_8_) was obtained by dissolving 66.2 mg K_2_S_2_O_8_ (Sigma, Poole, Missouri) in 100 mL distilled water. Solution B, 7 mM ABTS (2,2′-Azino-bis (3-ethylbenzothiazoline-6-sulfonic acid) diammonium salt) was prepared by dissolving 192 mg in 50 ml of distilled water. Solutions A and B were mixed in proportion of 1:9 (v/v) and left in the dark at room temperature overnight to generate the ABTS working solution. Prior to use, ABTS working solution was diluted and adjusted to an absorbance of 0.7±0.02 at λ 760nm using 5 mM phosphate buffer solution (pH=7.4), which was prepared by mixing 4.5g sodium chloride (NaCl), 0.1839g sodium phosphate monobasic (NaH_2_PO_4_·H_2_O) and 0.3677g sodium phosphate dibasic dodecahydrate (Na2HPO4·12H2O) in 500 mL of distilled water. **Analysis**: 10 µL of Trolox standards, samples and 50% methanol with distilled water as blank were mixed with290 µL of TEAC working solution in a 96-well microplate. The decrease in absorbance was measured at 734nm after 6 min incubation at 37 ⁰C. Each standard solution and sample solution was run in duplicate. The final results were expressed as µmol Trolox equivalent (TE)/g flour (DW).

**FRAP assay**, 0.278g Ferrous Sulphate (FeSO_4_.7H_2_O) were dissolved in 1L distilled water is as the standard stock solution. This was diluted to 278, 139, 69.5, 34.75, 17.375, 8.6875 and 4.34375 µg/mL by serial dilution for the standard calibration curve. **FRAP working solution preparation**: Solution A: acetate buffer (pH 3.6) was prepared by dissolving 3.1g sodium acetate trihydrate (CH_3_COONa_3_H_2_O) in a 100 mL flask using about 50 mL distilled water, this was mixed gently with16 mL concentrated acetic acid (CH_3_COOH), then made up to volume with distilled water. Solution B: 10mM 2,4,6-Tris(2-pyridyl)-s-triazine (TPTZ, C_18_H_12_N_6_), was prepared by dissolving 0.0781 TPTZ I in 25 mL of 40mM HCl. Solution C: 20mM ferric chloride (FeCl_3_.6H_2_O), was prepared by dissolving 0.5406 g FeCl_3_.6H_2_O in 100 mL H_2_O. The FRAP working solution was prepared by mixing solution A, B, C in proportion of 10:1:1. Fresh FRAP working solution was prepared before each assay. **Analysis**: 10µL of ferrous sulphate standards, extraction samples and distilled water as blank were mixed with 300µL of FRAP working reagent in the 96-well microplate and incubated at 37⁰C for 4 minutes. The absorbance of samples was measured at λ 593nm after incubation. Each standard solution and sample solution were run in duplicate. The final results were expressed as µmol Fe^2+^ equivalent/g flour (DW).

# **Results**

| ***Table S1*.**  Number (n) of wheat flour brands sampled and analysed for antioxidant activity and phenolic concentrations (A&P) and mineral content (MIN) in the retail survey in the UK and Germany in 2015 and 2016. | | | | | | | | | | |
| --- | --- | --- | --- | --- | --- | --- | --- | --- | --- | --- |
|  |  |  | **No. of flour samples** | | | | | | | |
| **Wheat species** | **Farming** | **Flour** | **2015** | |  | **2016** | |  | **TOTAL** | |
| **Country** | **system** | **type** | **A&P** | **MIN** |  | **A&P** | **MIN** |  | **A&P** | **MIN** |
| **Common** |  |  |  |  |  |  |  |  |  |  |
| **wheat flour** |  |  |  |  |  |  |  |  |  |  |
| **Germany** | **Conventional** | **White** | 0 | 0 |  | 12 | 12 |  | 12 | 12 |
|  |  | **Whole-grain** | 0 | 0 |  | 3 | 3 |  | 3 | 3 |
|  | **Organic** | **White** | 0 | 0 |  | 9 | 9 |  | 9 | 9 |
|  |  | **Whole-grain** | 0 | 0 |  | 6 | 6 |  | 6 | 6 |
| **UK** | **Conventional** | **White** | 12 | 12 |  | 15 | 15 |  | 27 | 27 |
|  |  | **Whole-grain** | 8 | 8 |  | 11 | 10** |  | 19 | 18 |
|  | **Organic** | **White** | 7 | 7 |  | 11 | 11* |  | 18 | 18 |
|  |  | **Whole-grain** | 8 | 7 |  | 10 | 10* |  | 18 | 17 |
|  | **TOTAL common wheat** | | **35** | **34** |  | **77** | **76** |  | **112** | **110** |
|  |  | |  |  |  |  |  |  |  |  |
| **Spelt**  **Wheat flour** |  |  |  |  |  |  |  |  |  |  |
| **Germany** | **Conventional** | **White** | 4 | 4 |  | 7 | 7 |  | 11 | 11 |
|  |  | **Whole-grain** | 2 | 2 |  | 3 | 3 |  | 5 | 5 |
|  | **Organic** | **White** | 2 | 2 |  | 4 | 4 |  | 6 | 6 |
|  |  | **Whole-grain** | 2 | 2 |  | 5 | 6 |  | 7 | 8 |
| **UK** | **Conventional** | **White** | 1 | 1 |  | 0 | 0 |  | 1 | 1 |
|  |  | **Whole-grain** | 1 | 1 |  | 5 | 5 |  | 6 | 6 |
|  | **Organic** | **White** | 2 | 2 |  | 4 | 3 |  | 6 | 5 |
|  |  | **Whole-grain** | 6 | 6 |  | 7 | 7 |  | 13 | 13 |
|  | **TOTAL spelt wheat** | | **20** | **20** |  | **35** | **34** |  | **55** | **55** |
|  |  | |  |  |  |  |  |  |  |  |
| **TOTAL NUMBER OF FLOUR SAMPLES** | | | **55** | **54** |  | **112** | **110** |  | **167** | **165** |
| *, includes one sample of self-rising flour; **, includes two samples of self-rising flour; data for Na, P, and Ca content obtained for self- rising flour were excluded from the statistical analyses of mineral composition data | | | | | | | | | | |

| ***Table S2*.** Interactions means ± SE for the effects of wheat species and farming system on copper concentrations in flour collected from UK and DE in 2015 and 2016 (results are expressed on a flour dry weight basis). | | | |
| --- | --- | --- | --- |
|  |  | **Factor 2** | |
|  | **Factor 1** | **farming system** | |
| **Parameters assessed** | **wheat species** | Organic | conventional |
|  |  |  |  |
| **Sulphur** | spelt | 0.94 ±0.05 **B a** | 1.05 ±0.05 **A a** |
| mg g^-1^ | common | 0.80 ±0.03 **A b** | 0.74 ±0.03 **B b** |
|  |  |  |  |
| **Copper** | spelt | 5.8 ±0.6 **A a** | 5.2 ±0.5 **A a** |
| mg kg^-1^ | common | 5.0 ±0.3 **A a** | 3.2 ±0.2 **B b** |
|  |  |  |  |
| **Aluminium** | spelt | 3.5 ±0.4 **A b** | 4.1±0.4 **A a** |
| mg kg^-1^ | common | 5.2 ±0.4 **A a** | 3.6±0.3 **B a** |
| For each parameter assessed means labelled with the same capital letter within the same row and same lower-case letter within the same column are not significant different (Tukey’s honestly significant difference test P<0.05) | | | |

***ANOVA 1: wheat species × farming system × flour type***

| ***Table S3*.** Main effect means ± SE and *p*-values for the effects and interaction between wheat species, farming system and flour type on phenolic content of flour collected from UK and DE between 2015 and 2016 (results are expressed on a flour dry weight basis) | | | | |
| --- | --- | --- | --- | --- |
|  | **Phenolics Content** | | | |
|  | **Free** | **Bound** | **Conjugated** | **Total** |
| **Factor** | µmol GAE g^-1^ flour (DW) | | | |
| **Species** |  |  |  |  |
| Spelt (n=55) | 4.9 ±0.2 | 3.9 ±0.4 | 0.7 ±0.1 | 9.4 ±0.4 |
| Wheat (n=112) | 4.6 ±0.1 | 3.4 ±0.3 | 0.6 ±0.1 | 8.5 ±0.4 |
| **Farming system** |  |  |  |  |
| Conventional (n=84) | 4.9 ±0.2 | 3.0 ±0.3 | 0.5 ±0.1 | 8.4 ±0.4 |
| Organic (n=83) | 4.4 ±0.1 | 4.1 ±0.3 | 0.7 ±0.1 | 9.2 ±0.4 |
| **Flour Type** |  |  |  |  |
| White (n=90) | 4.5 ±0.1 | 1.2 ±0.1 | 0.4 ±0.1 | 6.0 ±0.2 |
| Whole-grain (n=77) | 4.9 ±0.2 | 6.3 ±0.2 | 0.9 ±0.1 | 12.1 ±0.3 |
| **ANOVA** (p-values) |  |  |  |  |
| ***Main Effects*** |  |  |  |  |
| Species (SP) | **0.0176** | **0.0330** | **0.0386** | **0.0053** |
| Farming system (FS) | **0.0037** | **<0.0001** | **0.0001** | **0.0232** |
| Flour Type (FT) | **0.0024** | **<0.0001** | **<0.0001** | **<0.0001** |
| ***Interactions*** |  |  |  |  |
| **SP × FS** | NS | NS | NS | NS |
| **SP × FT** | **0.0001 ^1^** | **0.0022 ^1^** | **0.0004 ^1^** | **<0.0001 ^1^** |
| **FS × FT** | NS | *0.0690* | NS | *0.0742* |
| **SP × FS × FT** | NS | NS | NS | NS |
| **^1^**See table S3.1 for Interaction means ± SE; | | | | |

| ***Table S3.1*.** Interactions means ± SE for the effects of wheat species and flour type on phenolic content in flour collected from UK and DE between 2015 and 2016 (results are expressed on a flour dry weight basis) | | | |
| --- | --- | --- | --- |
| **Folin** | **Factor 1** | **Factor 2** | |
|  |  | **flour type** | |
| **Fraction** | **wheat species** | White | Whole-grain |
| **Free** | spelt | 5.1 ±0.3 **A a** | 4.7 ±0.2 **A a** |
| µmol GAE g^-1^ flour (DW) | wheat | 4.3 ±0.1 **B b** | 5.1 ±0.3 **A a** |
| **Bound** | spelt | 1.5 ±0.3 **B a** | 5.8 ±0.3 **A b** |
| µmol GAE g^-1^ flour (DW) | wheat | 1.1 ±0.1 **B a** | 6.7 ±0.3 **A a** |
| **Conjugated** | spelt | 0.5 ±0.1 **B b** | 0.8 ±0.1 **A b** |
| µmol GAE g^-1^ flour (DW) | wheat | 0.3 ±0.1 **B a** | 0.9 ±0.1 **A a** |
| **Total** | spelt | 7.1 ±0.4 **B a** | 11.2 ±0.4 **A b** |
| µmol GAE g^-1^ flour (DW) | wheat | 5.6 ±0.2 **B b** | 12.7 ±0.4 **A a** |
| For each parameter assessed means labelled with the same capital letter within the same row and same lower-case letter within the same column are not significant different (Tukey’s honestly significant difference test P<0.05) | | | |

| ***Table S4*.** Main effect means ± SE and *p*-values for the effects and interaction between wheat species, farming system and flour type on Antioxidant activity by FRAP of flour collected from UK and DE between 2015 and 2016 (results are expressed on a flour dry weight basis) | | | | |
| --- | --- | --- | --- | --- |
|  | **Antioxidant Activity by FRAP** | | | |
|  | **Free** | **Bound** | **Conjugated** | **Total** |
|  | µmol FeSO_4_ 7H_2_O g^-1^ flour (DW) | | | |
| **Species** |  |  |  |  |
| Spelt (n=55) | 1.2 ±0.1 | 3.3 ±0.3 | 0.6 ±0.1 | 5.1 ±0.4 |
| Wheat (n=112) | 1.2 ±0.1 | 3.0 ±0.3 | 0.7 ±0.1 | 4.8 ±0.4 |
| **Farming system** |  |  |  |  |
| Conventional (n=84) | 1.0 ±0.1 | 2.7 ±0.26 | 0.6 ±0.1 | 4.3 ±0.4 |
| Organic (n=83) | 1.4 ±0.1 | 3.6 ±0.29 | 0.7 ±0.1 | 5.5 ±0.4 |
| **Flour Type** |  |  |  |  |
| White (n=90) | 0.7 ±0.1 | 1.0 ±0.2 | 0.4 ±0.1 | 2.0 ±0.1 |
| Whole-grain (n=77) | 1.7 ±0.1 | 5.6 ±0.3 | 1.0 ±0.1 | 8.3 ±0.2 |
| **ANOVA** (p-values) |  |  |  |  |
| ***Main Effects*** |  |  |  |  |
| Species (SP) | NS | **0.0306** | NS | NS |
| Farming system (FS) | **0.0005** | **<0.0001** | NS | **<0.0001** |
| Flour Type (FT) | **<0.0001** | **<0.0001** | **<0.0001** | **<0.0001** |
| **Interactions** |  |  |  |  |
| **SP × FS** | NS | NS | NS | NS |
| **SP × FT** | **0.0391 ^1^** | **0.0005 ^1^** | NS | **0.0002 ^1^** |
| **FS × FT** | **0.0256 ^2^** | **0.0379 ^2^** | NS | **0.0131 ^2^** |
| **SP × FS × FT** | NS | NS | NS | NS |
| **^1^**See table S4.1 for Interaction means ± SE; **^2^**See table S4.2 for Interaction means ± SE; | | | | |

| ***Table S4.1.*** Interactions means ± SE for the effects of wheat species and flour type on antioxidant activity by FRAP of flour collected from UK and DE between 2015 and 2016 (results are expressed on a flour dry weight basis) | | | |
| --- | --- | --- | --- |
| **FRAP** | **Factor 1** | **Factor 2** | |
|  |  | **flour type** | |
| **Fraction** | **wheat species** | White | Whole-grain |
| **Free** | spelt | 0.7 ±0.1 **B a** | 1.6 ±0.1 **A a** |
| µmol FeSO_4_ 7H_2_O g^-1^ flour (DW) | wheat | 0.7 ±0.1 **B a** | 1.9 ±0.1 **A b** |
| **Bound** | spelt | 1.1 ±0.2 **B a** | 5.1 ±0.3 **A b** |
| µmol FeSO_4_ 7H_2_O g^-1^ flour (DW) | wheat | 0.9 ±0.1 **B a** | 6.0 ±0.2 **A a** |
| **Total** | spelt | 2.2 ±0.3 **B a** | 7.4 ±0.3 **A b** |
| µmol FeSO_4_ 7H_2_O g^-1^ flour (DW) | wheat | 1.9 ±0.1 **B a** | 8.9 ±0.3 **A a** |
| For each parameter assessed means labelled with the same capital letter within the same row and same lower-case letter within the same column are not significant different (Tukey’s honestly significant difference test P<0.05) | | | |

| ***Table S4.2.*** Interactions means ± SE for the effects of farming system and flour type on antioxidant activity by FRAP of flour collected from UK and DE between 2015 and 2016 (results are expressed on a flour dry weight basis) | | | |
| --- | --- | --- | --- |
| **FRAP** | **Factor 1** | **Factor 2** | |
|  |  | **farming system** | |
| **Fraction** | **flour Type** | organic | Conventional |
| **Free** | White | 0.7 ±0.1 **A b** | 0.7 ±0.1 **A b** |
| µmol FeSO_4_ 7H_2_O g^-1^ flour (DW) | Whole-grain | 1.9 ±0.1 **A a** | 1.5 ±0.1 **B a** |
| **Bound** | White | 1.0 ±0.1 **A b** | 1.0 ±0.1 **A b** |
| µmol FeSO_4_ 7H_2_O g^-1^ flour (DW) | Whole-grain | 5.9 ±0.2 **A a** | 5.3 ±0.2 **B a** |
| **Total** | White | 2.0 ±0.2 **A b** | 2.0 ±0.2 **A b** |
| µmol FeSO_4_ 7H_2_O g^-1^ flour (DW) | Whole-grain | 8.7 ±0.3 **A a** | 7.8 ±0.3 **B a** |
| For each parameter assessed means labelled with the same capital letter within the same row and same lower-case letter within the same column are not significant different (Tukey’s honestly significant difference test P<0.05) | | | |

| ***Table S5*.** Main effect means ± SE and *p*-values for the effects and interaction between wheat species, farming system and flour type on Antioxidant activity by TEAC of flour collected from UK and DE between 2015 and 2016 (results are expressed on a flour dry weight basis) | | | | |
| --- | --- | --- | --- | --- |
|  | **Antioxidant Activity by TEAC** | | | |
|  | **Free** | **Bound** | **Conjugated** | **Total** |
|  | µmol Trolox g^-1^ flour (DW) | | | |
| **Species** |  |  |  |  |
| Spelt (n=55) | 2.0 ±0.2 | 6.5 ±0.6 | 1.2 ±0.2 | 9.8 ±0.8 |
| Wheat (n=112) | 1.9 ±0.1 | 5.6 ±0.5 | 1.0 ±0.2 | 8.5 ±0.6 |
| **Farming system** |  |  |  |  |
| Conventional (n=84) | 1.8 ±0.1 | 5.1 ±0.5 | 0.9 ±0.2 | 7.8 ±0.6 |
| Organic (n=83) | 2.0 ±0.1 | 6.7 ±0.5 | 1.3 ±0.3 | 10.1 ±0.7 |
| **Flour Type** |  |  |  |  |
| White (n=90) | 1.5 ±0.1 | 2.0 ±0.1 | 0.4 ±0.1 | 3.9 ±0.2 |
| Whole-grain (n=77) | 2.4 ±0.1 | 10.5 ±0.3 | 1.9 ±0.3 | 14.8 ±0.4 |
| **ANOVA** (p-values) |  |  |  |  |
| ***Main Effects*** |  |  |  |  |
| Species (SP) | NS | **0.0152** | NS | **0.0380** |
| Farming system (FS) | NS | **<0.0001** | NS | **<0.0001** |
| Flour Type (FT) | **<0.0001** | **<0.0001** | **<0.0001** | **<0.0001** |
| **Interactions** |  |  |  |  |
| **SP × FS** | NS | NS | NS | NS |
| **SP × FT** | NS | **0.0045 ^1^** | 0.5488 | **0.0112 ^1^** |
| **FS × FT** | *0.0824* | NS | NS | *0.0750* |
| **SP × FS × FT** | NS | NS | NS | NS |
| **^1^**See table S5.1 for Interaction means ± SE; | | | | |

| ***Table S5.1.*** Interactions means ± SE for the effects of wheat species and flour type on antioxidant activity by TEAC of flour collected from UK and DE between 2015 and 2016 (results are expressed on a flour dry weight basis) | | | |
| --- | --- | --- | --- |
| **TEAC** | **Factor 1** | **Factor 2** | |
|  |  | **flour Type** | |
| **Fraction** | **wheat species** | White | Whole-grain |
| **Bound** | spelt | 2.4 ±0.4 **B a** | 9.6 ±0.6 **A b** |
| µmol Trolox g^-1^ flour (DW) | wheat | 1.9 ±0.1 **B a** | 11.0 ±0.4 **A a** |
| **Total** | spelt | 4.5 ±0.5 **B a** | 13.8 ±0.6 **A b** |
| µmol Trolox g^-1^ flour (DW) | wheat | 3.7 ±0.2 **B a** | 15.4 ±0.6 **A a** |
| For each parameter assessed means labelled with the same capital letter within the same row and same lower-case letter within the same column are not significant different (Tukey’s honestly significant difference test P<0.05) | | | |

| ***Table S6.*** Main effect means ± SE and *p*-values for the effects and interaction between wheat species, farming system and flour type on flavonoid content in flour collected from UK and DE between 2015 and 2016 (results are expressed on a flour dry weight basis) | | | | |
| --- | --- | --- | --- | --- |
|  | **Flavonoid Content** | | | |
|  | **Free** | **Bound** | **Conjugated** | **Total** |
|  | µmol Catechin g^-1^ flour (DW) | | | |
| **Species** |  |  |  |  |
| Spelt (n=55) | 0.2 ±0.1 | 0.6 ±0.1 | 0.13 ±0.03 | 1.0 ±0.1 |
| Wheat (n=112) | 0.3 ±0.1 | 0.6 ±0.1 | 0.11 ±0.02 | 1.0 ±0.1 |
| **Farming system** |  |  |  |  |
| Conventional (n=84) | 0.2 ±0.1 | 0.5 ±0.1 | 0.08 ±0.01 | 0.8 ±0.1 |
| Organic (n=83) | 0.3 ±0.1 | 0.7 ±0.1 | 0.15 ±0.03 | 1.1 ±0.1 |
| **Flour Type** |  |  |  |  |
| White (n=90) | 0.3 ±0.05 | 0.2 ±0.03 | 0.09 ±0.02 | 0.6 ±0.1 |
| Whole-grain (n=77) | 0.2 ±0.01 | 1.0 ±0.06 | 0.15 ±0.02 | 1.4 ±0.1 |
| **ANOVA** (p-values) |  |  |  |  |
| ***Main Effects*** |  |  |  |  |
| Species (SP) | NS | NS | NS | NS |
| Farming system (FS) | NS | *0.0704* | *0.0566* | **0.0094** |
| Flour Type (FT) | NS | **<0.0001** | **0.0011** | **<0.0001** |
| **Interactions** |  |  |  |  |
| **SP × FS** | NS | NS | NS | NS |
| **SP × FT** | NS | NS | NS | NS |
| **FS × FT** | NS | NS | **0.0022 ^1^** | NS |
| **SP × FS × FT** | NS | *0.0697* | NS | **0.0198 ^2^** |
| **^1^**See table S6.1 for Interaction means ± SE; **^2^**See table S6.2 for the interaction means ± SE. | | | | |

| ***Table S6.1.*** Interactions means ± SE for the effects of farming system and flour type on antioxidant activity by flavonoid in flour collected from UK and DE between 2015 and 2016 (results are expressed on a flour dry weight basis) | | | |
| --- | --- | --- | --- |
| **Flavonoid** | **Factor 1** | **Factor 2** | |
|  |  | **farming system** | |
| **Fraction** | **flour type** | organic | conventional |
| **Conjugated** | White | 0.1 ±0.1 **A a** | 0.1 ±0.1 **A a** |
| µmol Catechin g^-1^ flour (DW) | Whole-grain | 0.2 ±0.1 **A a** | 0.1 ±0.1 **A a** |
| For each parameter assessed means labelled with the same capital letter within the same row and same lower-case letter within the same column are not significant different (Tukey’s honestly significant difference test P<0.05) | | | |

| ***Table S6.2.*** Interactions means ± SE for the effects of wheat species, farming system and flour type on flavonoid content in bound fraction of flour collected from UK and DE between 2015 and 2016 (results are expressed on a flour dry weight basis) | | | | |
| --- | --- | --- | --- | --- |
| **Flavonoid** | **Factor 1** | **Factor 2** | **Factor 3** | |
|  |  |  | **farming system** | |
| **Fraction** | **wheat species** | **flour type** | organic | conventional |
| **Total** | spelt | White | 0.5 ±0.1 **A b** | 0.6 ±0.1 **A b** |
|  |  | Whole-grain | 1.5 ±0.1 **A a** | 1.1 ±0.1 **A a** |
| µmol Catechin g^-1^ flour (DW) | wheat | White | 0.8 ±0.2 **A b** | 0.5 ±0.1 **B b** |
|  |  | Whole-grain | 1.5 ±0.1 **A a** | 1.5 ±0.2 **A a** |
| For each parameter assessed means labelled with the same capital letter within the same row and same lower-case letter within the same column are not significant different (Tukey’s honestly significant difference test P<0.05) | | | | |

| ***Table S7.*** Main effect means ± SE and *p*-values for the effects and interaction between wheat species, farming system and flour type on ferulic acid detected by HPLC in flour collected from UK and DE between 2015 and 2016 (results are expressed on a flour dry weight basis) | | | | |
| --- | --- | --- | --- | --- |
|  | **Ferulic Acid (HPLC)** | | | |
|  | **Free** | **Bound** | **Conjugated** | **Total** |
|  | µmol/g flour (DW) | | | |
| **Species** |  |  |  |  |
| Spelt (n=55) | 0.8 ±0.1 | 355 ±33 | 13.5 ±0.9 | 370 ±34 |
| Wheat (n=112) | 0.9 ±0.1 | 338 ±29 | 12.5 ±0.7 | 352 ±30 |
| **Farming system** |  |  |  |  |
| Conventional (n=84) | 0.8 ±0.1 | 322 ±34 | 10.9 ±0.7 | 334 ±34 |
| Organic (n=83) | 0.9 ±0.1 | 367 ±30 | 14.8 ±0.9 | 382 ±31 |
| **Flour Type** |  |  |  |  |
| White (n=90) | 0.5 ±0.1 | 113 ± 8 | 7.2 ±0.4 | 120 ± 8 |
| Whole-grain (n=77) | 1.3 ±0.1 | 615 ±23 | 19.4 ±0.6 | 636 ±23 |
| **ANOVA** (p-values) |  |  |  |  |
| ***Main Effects*** |  |  |  |  |
| Species (SP) | **0.0164** | NS | NS | NS |
| Farming system (FS) | *0.0855* | NS | **<.0001** | **0.0887** |
| Flour Type (FT) | **<.0001** | **<.0001** | **<.0001** | **<.0001** |
| ***Interactions*** |  |  |  |  |
| SP × FS | NS | NS | NS | NS |
| SP × FT | *0.0861* | **0.0002 ^1^** | *0.0613* | **0.0002 ^1^** |
| FS × FT | NS | NS | **0.0034 ^2^** | NS |
| SP × FS × FT | NS | **0.0052 ^3^** | NS | **0.0062 ^3^** |
| **^1^**See table S7.1 for Interaction means ± SE; **^2^**See table S7.2 for Interaction means ± SE; **^3^**See table S7.3 for Interaction means ± SE | | | | |

| ***Table S7.1.*** Interactions means ± SE for the effects of wheat species and flour type on ferulic phenolic acid concentration detected by HPLC in flour collected from UK and DE between 2015 and 2016 (results are expressed on a flour dry weight basis) | | | |
| --- | --- | --- | --- |
|  | **Factor 1** | **Factor 2** | |
|  |  | **flour type** | |
| **Fraction** | **wheat species** | White | Whole-grain |
|  |  | **Ferulic Acid (HPLC)** | |
| **Bound** | spelt | 123 ±24 **B a** | 535 ±26 **A a** |
| µmol g^-1^ flour (DW) | wheat | 109 ± 7 **B a** | 669 ±32 **A b** |
| **Total** | spelt | 131 ±24 **B a** | 554 ±26 **A b** |
| µmol g^-1^ flour (DW) | wheat | 117 ± 7 **B a** | 691 ±32 **A a** |
| For each parameter assessed means labelled with the same capital letter within the same row and same lower-case letter within the same column are not significant different (Tukey’s honestly significant difference test P<0.05) | | | |

| ***Table S7.2.*** Interactions means ± SE for the effects of farming system and flour type on ferulic acid concentration detected by HPLC in flour collected from UK and DE between 2015 and 2016 (results are expressed on a flour dry weight basis) | | | |
| --- | --- | --- | --- |
|  | **Factor 1** | **Factor 2** | |
|  |  | **flour type** | |
| **Fraction** | **farming system** | White | Whole-grain |
|  |  | **Ferulic Acid (HPLC)** | |
| **conjugated** | conventional | 6.9 ±0.6 **B a** | 17.0 ±0.7 **A b** |
| µmol g^-1^ flour (DW) | organic | 7.6 ±0.5 **B a** | 21.1 ±0.8 **A a** |
| For each parameter assessed means labelled with the same capital letter within the same row and same lower-case letter within the same column are not significant different (Tukey’s honestly significant difference test P<0.05) | | | |

| ***Table S7.3*.** Interactions means ± SE for the effects of wheat species, flour type and farming system on ferulic phenolic acid concentration detected by HPLC in flour collected from UK and DE between 2015 and 2016 (results are expressed on a flour dry weight basis) | | | | |
| --- | --- | --- | --- | --- |
|  | **Factor 1** | **Factor 2** | **Factor 3** | |
|  |  |  | **flour type** | |
| **Fraction** | **species** | **farming system** | White | Whole-grain |
|  |  |  | **Ferulic Acid (HPLC)** | |
| **Bound** | spelt | conventional | 151 ±44 **B a** | 492 ±57 **A c** |
|  |  | organic | 95 ±15 **B a** | 559 ±25 **A bc** |
| µmol g^-1^ flour (DW) | wheat | conventional | 105 ± 9 **B a** | 716 ±52 **A a** |
|  |  | organic | 115 ±11 **B a** | 626 ±36 **A b** |
| **Total** | spelt | conventional | 159 ±45 **B a** | 509 ±58 **A c** |
|  |  | organic | 103 ±16 **B a** | 579 ±25 **A bc** |
| µmol g^-1^ flour (DW) | wheat | conventional | 112 ± 9 **B a** | 735 ±52 **A a** |
|  |  | organic | 123 ±12 **B a** | 650 ±36 **A b** |
| For each parameter assessed means labelled with the same capital letter within the same row and same lower-case letter within the same column are not significant different (Tukey’s honestly significant difference test P<0.05) | | | | |

| ***Table S8.*** Main effect means ± SE and *p*-values for the effects and interaction between wheat species, farming system and flour type on Sinapic acids detected by HPLC in flour collected from UK and DE between 2015 and 2016 (results are expressed on a flour dry weight basis) | | | | |
| --- | --- | --- | --- | --- |
|  | **Sinapic Acid (HPLC)** | | | |
|  | **Free** | **Bound** | **Conjugated** | **Total** |
|  | µmol g^-1^ flour (DW) | | | |
| **Species** |  |  |  |  |
| Spelt (n=55) | 5.5 ±0.7 | 8.0 ±0.9 | 14.8 ±1.4 | 28.3 ±2.6 |
| Wheat (n=112) | 4.3 ±0.5 | 9.4 ±1.0 | 14.9 ±1.1 | 28.6 ±2.4 |
| **Farming system** |  |  |  |  |
| Conventional (n=84) | 3.7 ±0.5 | 8.9 ±1.2 | 13.4 ±1.2 | 26.0 ±2.6 |
| Organic (n=83) | 5.7 ±0.6 | 9.0 ±0.9 | 16.4 ±1.3 | 31.1 ±2.5 |
| **Flour Type** |  |  |  |  |
| White (n=90) | 2.8 ±0.4 | 2.0 ±0.3 | 6.8 ±0.7 | 11.6 ±1.2 |
| Whole-grain (n=77) | 6.9 ±0.7 | 17.1 ±0.9 | 24.3 ±1.0 | 48.3 ±1.9 |
| **ANOVA** (p-values) |  |  |  |  |
| ***Main Effects*** |  |  |  |  |
| Species (SP) | **0.0432** | NS | NS | NS |
| Farming system (FS) | **0.0029** | NS | **0.0138** | **0.0299** |
| Flour Type (FT) | **<0.0001** | **<0.0001** | **<0.0001** | **<0.0001** |
| ***Interactions*** |  |  |  |  |
| SP × FS | NS | NS | NS | NS |
| SP × FT | 0.0331 | **<0.0001 ^1^** | **0.0011 ^1^** | **<0.0001 ^1^** |
| FS × FT | NS | *0.0903* | *0.0612* | NS |
| SP × FS × FT | NS | **0.0166 ^2^** | NS | NS |
| **^1^**See table S8.1 for Interaction means ± SE; **^2^**See table S8.2 for Interaction means ± SE; | | | | |

| ***Table S8.1*.** Interactions means ± SE for the effects of wheat species and flour type on sinapic acid concentration detected by HPLC in flour collected from UK and DE between 2015 and 2016 (results are expressed on a flour dry weight basis) | | | |
| --- | --- | --- | --- |
|  | **Factor 1** | **Factor 2** | |
|  |  | **flour type** | |
| **Fraction** | **wheat species** | White | Whole-grain |
|  |  | **Sinapic Acid (HPLC)** | |
| **Free** | spelt | 3.9 ±0.8 **B a** | 6.8 ±1.0 **A a** |
| µmol g^-1^ flour (DW) | wheat | 2.4 ±0.4 **B b** | 7.0 ±0.9 **A a** |
| **Conjugated** | spelt | 7.6 ±2.0 **B a** | 20.4 ±1.3 **A b** |
| µmol g^-1^ flour (DW) | wheat | 6.5 ±0.6 **B a** | 26.9 ±1.2 **A a** |
| **Total** | spelt | 14.0 ±3.3 **B a** | 39.4 ±2.4 **A b** |
| µmol g^-1^ flour (DW) | wheat | 10.7 ±1.1 **B a** | 54.3 ±2.5 **A a** |
| For each parameter assessed means labelled with the same capital letter within the same row and same lower-case letter within the same column are not significant different (Tukey’s honestly significant difference test P<0.05) | | | |

| ***Table S8.2*.** Interactions means ± SE for the effects of wheat species, flour type and farming system on sinapic acid concentration in flour collected from UK and DE between 2015 and 2016 (results are expressed on a flour dry weight basis) | | | | |
| --- | --- | --- | --- | --- |
|  | **Factor 1** | **Factor 2** | **Factor 3** | |
|  |  |  | **flour type** | |
| **Fraction** | **wheat species** | **farming system** | White | Whole-grain |
|  |  |  | **Sinapic Acid (HPLC)** | |
| **Bound** | spelt | conventional | 3.6 ±1.8 **B a** | 12.4 ±1.7 **A c** |
|  |  | organic | 1.6 ±0.4 **B a** | 12.1 ±0.9 **A c** |
| µmol g^-1^ flour (DW) | wheat | conventional | 1.7 ±0.2 **B a** | 22.9 ±1.9 **A a** |
|  |  | organic | 1.9 ±0.3 **B a** | 18.0 ±1.3 **A b** |
| For each parameter assessed means labelled with the same capital letter within the same row and same lower-case letter within the same column are not significant different (Tukey’s honestly significant difference test P<0.05) | | | | |

| ***Table S9*.** Main effect means ± SE and *p*-values for the effects and interaction between wheat species, farming system and flour type on 4-hydroxybenzoic acid concentration detected by HPLC in flour collected from UK and DE between 2015 and 2016 (results are expressed on a flour dry weight basis) | | | | |
| --- | --- | --- | --- | --- |
|  | **4-hydroxybenzoic acid (HPLC)** | | | |
|  | **Free** | **Bound** | **Conjugated** | **Total** |
| **Factor** | µmol/g flour (DW) | | | |
| **Species** |  |  |  |  |
| Spelt (n=55) | 0.98 ±0.05 | 2.12 ±0.19 | 2.34 ±0.15 | 5.45 ±0.36 |
| Wheat (n=112) | 1.08 ±0.06 | 1.95 ±0.16 | 1.98 ±0.10 | 5.01 ±0.30 |
| **Farming system** |  |  |  |  |
| Conventional (n=84) | 1.03 ±0.06 | 1.90 ±0.19 | 1.83 ±0.10 | 4.76 ±0.33 |
| Organic (n=83) | 1.07 ±0.06 | 2.11 ±0.17 | 2.37 ±0.13 | 5.55 ±0.33 |
| **Flour Type** |  |  |  |  |
| White (n=90) | 0.73 ±0.04 | 0.70 ±0.06 | 1.30 ±0.06 | 2.73 ±0.13 |
| Whole-grain (n=77) | 1.42 ±0.05 | 3.53 ±0.12 | 3.03 ±0.09 | 7.98 ±0.19 |
| **ANOVA** (p-values) |  |  |  |  |
| ***Main Effects*** |  |  |  |  |
| Species (SP) | NS | NS | **0.0025** | **0.0444** |
| Farming system (FS) | NS | NS | **<.0001** | **0.0014** |
| Flour Type (FT) | **<.0001** | **<.0001** | **<.0001** | **<.0001** |
| ***Interactions*** |  |  |  |  |
| SP × FS | NS | NS | NS | NS |
| SP × FT | **<.0001 ^1^** | **0.0001** | NS | **0.0001** |
| FS × FT | NS | NS | **0.0269 ^2^** | NS |
| SP × FS × FT | NS | **0.0104 ^3^** | NS | **0.0376 ^3^** |
| **^1^**See table S9.1 for Interaction means ± SE; **^2^**See table S9.2 for Interaction means ± SE; **^3^**See table S9.3 for Interaction means ± SE | | | | |

| ***Table S9.1.*** Interactions means ± SE for the effects of wheat species and flour type on 4-hydroxybenzoic acid concentration detected by HPLC in flour collected from UK and DE between 2015 and 2016 (results are expressed on a flour dry weight basis) | | | |
| --- | --- | --- | --- |
|  | **Factor 1** | **Factor 2** | |
|  |  | **Flour Type** | |
| **Fraction** | **wheat species** | White | Whole-grain |
|  |  | **4-hydroxybenzoic acid (HPLC)** | |
| **Free** | spelt | 0.76 ±0.07 **B a** | 1.15 ±0.06 **A b** |
| µmol/g flour (DW) | wheat | 0.71 ±0.05 **B a** | 1.61 ±0.07 **A a** |
| For each parameter assessed means labelled with the same capital letter within the same row and same lower-case letter within the same column are not significant different (Tukey’s honestly significant difference test P<0.05) | | | |

| ***Table S9.2*.** Interactions means ± SE for the effects of farming system and flour type on 4-hydroxybenzoic acid concentration detected by HPLC in flour collected from UK and DE between 2015 and 2016 (results are expressed on a flour dry weight basis) | | | |
| --- | --- | --- | --- |
|  | **Factor 1** | **Factor 2** | |
|  |  | **Flour Type** | |
| **Fraction** | **farming system** | White | Whole-grain |
|  |  | **4-hydroxybenzoic acid (HPLC)** | |
| **Conjugated** | conventional | 1.25 ±0.08 **B a** | 2.72 ±0.09 **A b** |
| µmol/g flour (DW) | organic | 1.38 ±0.07 **B a** | 3.26 ±0.13 **A a** |
| For each parameter assessed means labelled with the same capital letter within the same row and same lower-case letter within the same column are not significant different (Tukey’s honestly significant difference test P<0.05) | | | |

| ***Table S9.3*.** Interactions means ± SE for the effects of wheat species, flour type and farming system on 4-hydroxybenzoic acid concentration detected by HPLC in flour collected from UK and DE between 2015 and 2016 (results are expressed on a flour dry weight basis) | | | | |
| --- | --- | --- | --- | --- |
|  | **Factor 1** | **Factor 2** | **Factor 3** | |
|  |  |  | **Flour Type** | |
| **Fraction** | **wheat species** | **farming system** | White | Whole-grain |
|  |  |  | **4-hydroxybenzoic acid (HPLC)** | |
| **Bound** | spelt | conventional | 1.09 ±0.36 **B a** | 2.93 ±0.36 **A c** |
|  |  | organic | 0.67 ±0.08 **B a** | 3.18 ±0.17 **A bc** |
| µmol/g flour (DW) | wheat | conventional | 0.62 ±0.04 **B b** | 4.10 ±0.25 **A a** |
|  |  | organic | 0.66 ±0.06 **B a** | 3.59 ±0.19 **A b** |
| **Total** | spelt | conventional | 3.44 ±0.65 **B a** | 6.72 ±0.56 **A b** |
|  |  | organic | 2.81 ±0.21 **B ab** | 7.54 ±0.36 **A b** |
| µmol/g flour (DW) | wheat | conventional | 2.51 ±0.17 **B b** | 8.48 ±0.30 **A a** |
|  |  | organic | 2.71 ±0.21 **B ab** | 8.47 ±0.31 **A a** |
| For each parameter assessed means labelled with the same capital letter within the same row and same lower-case letter within the same column are not significant different (Tukey’s honestly significant difference test P<0.05) | | | | |

| ***Table S10.*** Main effect means ± SE and *p*-values for the effects and interaction between wheat species, farming system and flour type on vanillic acid detected by HPLC in flour collected from UK and DE between 2015 and 2016 (results are expressed on a flour dry weight basis) | | | | |
| --- | --- | --- | --- | --- |
|  | **vanillic acid (HPLC)** | | | |
|  | **Free** | **Bound** | **Conjugated** | **Total** |
|  | µmol/g flour (DW) | | | |
| **Species** |  |  |  |  |
| Spelt (n=55) | 0.75 ±0.07 | 2.18 ±0.19 | 3.2 ±0.2 | 6.1 ±0.4 |
| Wheat (n=112) | 0.77±0.06 | 2.28 ±0.20 | 2.8 ±0.2 | 5.9 ±0.4 |
| **Farming system** |  |  |  |  |
| Conventional (n=84) | 0.73 ±0.07 | 2.13 ±0.22 | 2.6 ±0.2 | 5.5 ±0.4 |
| Organic (n=83) | 0.80 ±0.06 | 2.36 ±0.19 | 3.2 ±0.2 | 6.4 ±0.4 |
| **Flour Type** |  |  |  |  |
| White (n=90) | 0.33 ±0.02 | 0.73 ±0.05 | 1.7 ±0.1 | 2.7 ±0.2 |
| Whole-grain (n=77) | 1.27 ±0.05 | 4.03 ±0.14 | 4.4 ±0.1 | 9.7 ±0.3 |
| **ANOVA** (p-values) |  |  |  |  |
| ***Main Effects*** |  |  |  |  |
| Species (SP) | NS | NS | **0.0329** | NS |
| Farming system (FS) | NS | *0.0929* | **0.0003** | **0.0021** |
| Flour Type (FT) | **<.0001** | **<.0001** | **<.0001** | **<.0001** |
| ***Interactions*** |  |  |  |  |
| **SP × FS** | NS | NS | NS | NS |
| **SP × FT** | **0.0002 ^1^** | **<0.0001** | **0.0010 ^1^** | **<.0001 ^1^** |
| **FS × FT** | NS | NS | NS | NS |
| **SP × FS × FT** | NS | **0.0240 ^2^** | NS | NS |
| **^1^**See table S10.1 for Interaction means ± SE; **^2^**See table S10.2 for Interaction means ± SE; | | | | |

| ***Table S10.1*.** Interactions means ± SE for the effects of wheat species and flour type on vanillic acid concentration detected by HPLC in flour collected from UK and DE between 2015 and 2016 (results are expressed on a flour dry weight basis) | | | |
| --- | --- | --- | --- |
|  | **Factor 1** | **Factor 2** | |
|  |  | **flour Type** | |
| **Fraction** | **wheat species** | White | Whole-grain |
|  |  | **vanillic acid (HPLC)** | |
| **Free** | spelt | 0.35 ±0.04 **B a** | 1.06 ±0.07 **A b** |
| µmol/g flour (DW) | wheat | 0.33 ±0.02 **B a** | 1.41 ±0.07 **A a** |
| **Conjugated** | spelt | 2.03 ±0.19 **B a** | 4.01 ±0.16 **A b** |
| µmol/g flour (DW) | wheat | 1.55 ±0.10 **B b** | 4.59 ±0.17 **A a** |
| **Total** | spelt | 3.24 ±0.36 **B a** | 8.28 ±0.29 **A b** |
| µmol/g flour (DW) | wheat | 2.56 ±0.16 **B a** | 10.58 ±0.32 **A a** |
| For each parameter assessed means labelled with the same capital letter within the same row and same lower-case letter within the same column are not significant different (Tukey’s honestly significant difference test P<0.05) | | | |

| ***Table S10.2*.** Interactions means ± SE for the effects of wheat species, flour type and farming system on vanillic acid concentration detected by HPLC in flour collected from UK and DE between 2015 and 2016 (results are expressed on a flour dry weight basis) | | | | |
| --- | --- | --- | --- | --- |
|  | **Factor 1** | **Factor 2** | **Factor 3** | |
|  |  |  | **flour type** | |
| **Fraction** | **wheat Species** | **farming system** | White | Whole-grain |
|  |  |  | **vanillic acid (HPLC)** | |
| **Bound** | spelt | conventional | 1.02 ±0.30 **B a** | 3.09 ±0.34 **A c** |
|  |  | organic | 0.70 ±0.10 **B a** | 3.27 ±0.13 **A c** |
| µmol/g flour (DW) | wheat | conventional | 0.65 ±0.06 **B a** | 4.89 ±0.26 **A a** |
|  |  | organic | 0.71 ±0.08 **B a** | 4.29 ±0.23 **A b** |
| For each parameter assessed means labelled with the same capital letter within the same row and same lower-case letter within the same column are not significant different (Tukey’s honestly significant difference test P<0.05) | | | | |

| ***Table S11*.** Main effect means ± SE and *p*-values for the effects and interaction between wheat species, farming system and flour type on syringic acid detected by HPLC in flour collected from UK and DE between 2015 and 2016 (results are expressed on a flour dry weight basis) | | | | |
| --- | --- | --- | --- | --- |
|  | **syringic acid (HPLC)** | | | |
|  | **Free** | **Bound** | **Conjugated** | **Total** |
|  | µmol/g flour (DW) | | | |
| **Species** |  |  |  |  |
| Spelt (n=55) | 0.49 ±0.04 | 2.48 ±0.24 | 3.34 ±0.24 | 6.30 ±0.47 |
| Wheat (n=112) | 0.53 ±0.04 | 2.96 ±0.28 | 3.15 ±0.20 | 6.64 ±0.49 |
| **Farming system** |  |  |  |  |
| Conventional (n=84) | 0.45 ±0.04 | 2.86 ±0.33 | 2.89 ±0.22 | 6.20 ±0.55 |
| Organic (n=83) | 0.59 ±0.04 | 2.75 ±0.25 | 3.54 ±0.22 | 6.87 ±0.48 |
| **Flour Type** |  |  |  |  |
| White (n=90) | 0.30 ±0.02 | 0.78 ±0.06 | 1.71 ±0.09 | 2.79 ±0.16 |
| Whole-grain (n=77) | 0.77 ±0.04 | 5.18 ±0.25 | 4.96 ±0.18 | 10.91 ±0.36 |
| **ANOVA** (p-values) |  |  |  |  |
| ***Main Effects*** |  |  |  |  |
| Species (SP) | NS | **0.0463** | NS | NS |
| Farming system (FS) | **0.0028** | NS | **0.0013** | **0.0197** |
| Flour Type (FT) | **<.0001** | **<.0001** | **<.0001** | **<.0001** |
| ***Interactions*** |  |  |  |  |
| SP × FS | NS | NS | NS | NS |
| SP × FT | **0.0007 ^1^** | **<.0001** | **0.0054 ^1^** | **<.0001** |
| FS × FT | NS | NS | NS | NS |
| SP × FS × FT | NS | **0.0026 ^2^** | NS | **0.0094 ^2^** |
| **^1^**See table S11.1 for Interaction means ± SE; **^2^**See table S11.2 for Interaction means ± SE; | | | | |

| ***Table S11.1*.** Interactions means ± SE for the effects of wheat species and flour type on syringic acid concentration detected by HPLC in flour collected from UK and DE between 2015 and 2016 (results are expressed on a flour dry weight basis) | | | |
| --- | --- | --- | --- |
|  | **Factor 1** | **Factor 2** | |
|  |  | **flour Type** | |
| **Fraction** | **wheat species** | White | Whole-grain |
|  |  | **syringic acid (HPLC)** | |
| **Free** | spelt | 0.31 ±0.03 **B a** | 0.62 ±0.05 **A b** |
| µmol/g flour (DW) | wheat | 0.30 ±0.03 **B a** | 0.87 ±0.06 **A a** |
| **Conjugated** | spelt | 1.89 ±0.19 **B a** | 4.45 ±0.25 **A b** |
| µmol/g flour (DW) | wheat | 1.64 ±0.10 **B a** | 5.30 ±0.23 **A a** |
| For each parameter assessed means labelled with the same capital letter within the same row and same lower-case letter within the same column are not significant different (Tukey’s honestly significant difference test P<0.05) | | | |

| ***Table S11.2.*** Interactions means ± SE for the effects of wheat species, flour type and farming system on syringic acid concentration detected by HPLC in flour collected from UK and DE between 2015 and 2016 (results are expressed on a flour dry weight basis) | | | | |
| --- | --- | --- | --- | --- |
|  | **Factor 1** | **Factor 2** | **Factor 3** | |
|  |  |  | **flour Type** | |
| **Fraction** | **wheat species** | **farming system** | **White** | **Whole-grain** |
|  |  |  | **syringic acid (HPLC)** | |
| **Bound** | Spelt | conventional | 1.10 ±0.35 **B a** | 3.76 ±0.50 **A c** |
|  |  | organic | 0.54 ±0.06 **B a** | 3.79 ±0.18 **A c** |
| µmol/g flour (DW) | Wheat | conventional | 0.76 ±0.06 **B a** | 7.11 ±0.48 **A a** |
|  |  | organic | 0.77 ±0.09 **B a** | 5.22 ±0.35 **A b** |
| **Total** | Spelt | conventional | 3.52 ±0.73 **B a** | 8.42 ±0.82 **A c** |
|  |  | organic | 2.51 ±0.19 **B a** | 9.10 ±0.32 **A c** |
| µmol/g flour (DW) | Wheat | conventional | 2.56 ±0.20 **B a** | 13.00 ±0.72 **A a** |
|  |  | organic | 2.92 ±0.29 **B a** | 11.65 ±0.54 **A b** |
| For each parameter assessed means labelled with the same capital letter within the same row and same lower-case letter within the same column are not significant different (Tukey’s honestly significant difference test P<0.05) | | | | |

| ***Table S12*.** Main effect means ± SE and *p*-values for the effects and interaction between wheat species, farming system and flour type on p-coumaric acid concentration detected by HPLC in flour collected from UK and DE between 2015 and 2016 (results are expressed on a flour dry weight basis) | | | | |
| --- | --- | --- | --- | --- |
|  | **p-coumaric acid (HPLC)** | | | |
|  | **Free** | **Bound** | **Conjugated** | **Total** |
|  | µmol/g flour (DW) | | | |
| **Species** |  |  |  |  |
| Spelt (n=55) | 2.73 ±0.33 | 12.5 ±1.2 | 1.34 ±0.12 | 16.6 ±1.5 |
| Wheat (n=112) | 2.54 ±0.27 | 10.2 ±1.0 | 1.60 ±0.15 | 14.3 ±1.3 |
| **Farming system** |  |  |  |  |
| Conventional (n=84) | 2.15 ±0.26 | 9.1 ±1.0 | 0.98 ±0.09 | 12.3 ±1.2 |
| Organic (n=83) | 3.06 ±0.33 | 12.8 ±1.1 | 2.06 ±0.19 | 17.9 ±1.5 |
| **Flour Type** |  |  |  |  |
| White (n=90) | 1.56 ±0.19 | 3.7 ±0.3 | 0.84 ±0.09 | 6.1 ±0.5 |
| Whole-grain (n=77) | 3.83 ±0.35 | 19.4 ±0.9 | 2.30 ±0.18 | 25.6 ±1.2 |
| **ANOVA** (p-values) |  |  |  |  |
| ***Main Effects*** |  |  |  |  |
| Species (SP) | NS | **0.0227** | NS | **0.079** |
| Farming system (FS) | **0.0066** | **0.0005** | **<.0001** | **<.0001** |
| Flour Type (FT) | **<.0001** | **<.0001** | **<.0001** | **<.0001** |
| ***Interactions*** |  |  |  |  |
| SP × FS | NS | *0.0579* | **0.0027 ^1^** | **0.0406 ^1^** |
| SP × FT | **0.0061 ^2^** | *0.0815* | NS | **0.0163 ^2^** |
| FS × FT | NS | *0.0598* | **0.0055 ^3^** | **0.0278 ^3^** |
| SP × FS × FT | NS | NS | NS | NS |
| **^1^**See table S12.1 for Interaction means ± SE; **^2^**See table S12.2 for Interaction means ± SE; **^3^**See table S12.3 for Interaction means ± SE | | | | |

| ***Table S12.1*.** Interactions means ± SE for the effects of wheat specie and farming system on p-coumaric acid concentration detected by HPLC in flour collected from UK and DE between 2015 and 2016 (results are expressed on a flour dry weight basis) | | | |
| --- | --- | --- | --- |
|  | **Factor 1** | **Factor 2** | |
|  |  | **farming system** | |
| **Fraction** | **wheat species** | conventional | organic |
|  |  | **p-coumaric acid (HPLC)** | |
| **Conjugated** | spelt | 1.2 ±0.2 **A a** | 1.5 ±0.2 **A b** |
| µmol/g flour (DW) | wheat | 0.9 ±0.1 **B a** | 2.4 ±0.3 **A a** |
| **Total** | spelt | 15.3 ±2.6 **A a** | 17.6 ±1.8 **A a** |
| µmol/g flour (DW) | wheat | 11.1 ±1.3 **B a** | 18.1 ±2.1 **A a** |
| For each parameter assessed means labelled with the same capital letter within the same row and same lower-case letter within the same column are not significant different (Tukey’s honestly significant difference test P<0.05) | | | |

| ***Table S12.2*.** Interactions means ± SE for the effects of wheat specie and flour type on p-coumaric acid concentration detected by HPLC in flour collected from UK and DE between 2015 and 2016 (results are expressed on a flour dry weight basis) | | | |
| --- | --- | --- | --- |
|  | **Factor 1** | **Factor 2** | |
|  |  | **flour type** | |
| **Fraction** | **wheat species** | White | Whole-grain |
|  |  | **p-coumaric acid (HPLC)** | |
| **Free** | spelt | 1.93 ±0.37 **B a** | 3.35 ±0.49 **A a** |
| µmol/g flour (DW) | wheat | 1.42 ±0.21 **B a** | 4.15 ±0.49 **A a** |
| **Total** | spelt | 7.5 ±1.2 **B a** | 23.7 ±1.6 **A a** |
| µmol/g flour (DW) | wheat | 5.6 ±0.5 **B a** | 26.8 ±1.7 **A a** |
| For each parameter assessed means labelled with the same capital letter within the same row and same lower-case letter within the same column are not significant different (Tukey’s honestly significant difference test P<0.05) | | | |

| ***Table S12.3*.** Interactions means ± SE for the effects of farming system and flour type on p-coumaric acid concentration detected by HPLC in flour collected from UK and DE between 2015 and 2016 (results are expressed on a flour dry weight basis) | | | |
| --- | --- | --- | --- |
|  | **Factor 1** | **Factor 2** | |
|  |  | **flour type** | |
| **Fraction** | **farming system** | White | Whole-grain |
|  |  | **p-coumaric acid (HPLC)** | |
| **Conjugated** | conventional | 0.6 ±0.1 **B b** | 1.6 ±0.2 **A b** |
| µmol/g flour (DW) | organic | 1.2 ±0.2 **B a** | 2.8 ±0.3 **A a** |
| **Total** | conventional | 5.4 ±0.7 **B a** | 22.9 ±1.6 **A b** |
| µmol/g flour (DW) | organic | 7.0 ±0.7 **B a** | 27.6 ±1.7 **A a** |
| For each parameter assessed means labelled with the same capital letter within the same row and same lower-case letter within the same column are not significant different (Tukey’s honestly significant difference test P<0.05) | | | |

| ***Table S13*.** Main effect means ± SE and *p*-values for the effects and interaction between wheat species, farming system and flour type on syringaldeyde concentration detected by HPLC in flour collected from UK and DE between 2015 and 2016 (results are expressed on a flour dry weight basis) | | | | |
| --- | --- | --- | --- | --- |
|  | **Syringaldeyde** | | | |
|  | **Free** | **Bound** | **Conjugated** | **Total** |
|  | µmol/g flour (DW) | | | |
| **Species** |  |  |  |  |
| Spelt (n=55) | 0.19 ±0.02 | 4.16 ±0.41 | 1.47 ±0.12 | 5.8 ±0.5 |
| Wheat (n=112) | 0.41 ±0.07 | 4.28 ±0.40 | 1.31 ±0.09 | 6.0 ±0.5 |
| **Farming system** |  |  |  |  |
| Conventional (n=84) | 0.49 ±0.10 | 3.88 ±0.43 | 1.10 ±0.09 | 5.5 ±0.6 |
| Organic (n=83) | 0.19 ±0.02 | 4.60 ±0.41 | 1.62 ±0.11 | 6.4 ±0.5 |
| **Flour Type** |  |  |  |  |
| White (n=90) | 0.22 ±0.03 | 1.29 ±0.14 | 0.67 ±0.06 | 2.2 ±0.2 |
| Whole-grain (n=77) | 0.48 ±0.10 | 7.69 ±0.32 | 2.16 ±0.08 | 10.3 ±0.4 |
| **ANOVA** (p-values) |  |  |  |  |
| ***Main Effects*** |  |  |  |  |
| Species (SP) | *0.0568* | NS | *0.0786* | NS |
| Farming system (FS) | **0.0080** | **0.0499** | **<0.0001** | **0.0302** |
| Flour Type (FT) | **0.0004** | **<0.0001** | **<0.0001** | **<0.0001** |
| ***Interactions*** |  |  |  |  |
| SP × FS | *0.0552* | NS | *0.0973* | NS |
| SP × FT | *0.0835* | **<0.0001 ^1^** | NS | **<0.0001 ^1^** |
| FS × FT | *0.0777* | NS | **0.0433 ^2^** | NS |
| SP × FS × FT | NS | NS | NS | NS |
| **^1^**See table S13.1 for Interaction means ± SE; **^2^**See table S13.2 for Interaction means ± SE; | | | | |

| ***Table S13.1*.** Interactions means ± SE for the effects of wheat species and flour type on syringaldeyde concentration detected by HPLC in flour collected from UK and DE between 2015 and 2016 (results are expressed on a flour dry weight basis) | | | |
| --- | --- | --- | --- |
|  | **Factor 1** | **Factor 2** | |
|  |  | **flour Type** | |
| **Fraction** | **wheat species** | White | Whole-grain |
|  |  | **syringaldeyde (HPLC)** | |
| **Bound** | spelt | 1.70 ±0.42 **B a** | 6.05 ±0.38 **A b** |
| µmol/g flour (DW) | wheat | 1.14 ±0.11 **B a** | 8.79 ±0.41 **A a** |
| **Total** | spelt | 2.55 ±0.55 **B a** | 8.34 ±0.44 **A b** |
| µmol/g flour (DW) | wheat | 2.05 ±0.16 **B a** | 11.67 ±0.49 **A a** |
| For each parameter assessed means labelled with the same capital letter within the same row and same lower-case letter within the same column are not significant different (Tukey’s honestly significant difference test P<0.05) | | | |

| ***Table S13.2*.** Interactions means ± SE for the effects of wheat species and flour type on syringaldeyde concentration detected by HPLC in flour collected from UK and DE between 2015 and 2016 (results are expressed on a flour dry weight basis) | | | |
| --- | --- | --- | --- |
|  | **Factor 1** | **Factor 2** | |
|  |  | **flour type** | |
| **Fraction** | **farming system** | White | Whole-grain |
|  |  | **syringaldeyde (HPLC)** | |
| **Conjugated** | Conventional | 0.59 ±0.07 **B a** | 1.89 ±0.1 **A b** |
| µmol/g flour (DW) | Organic | 0.79 ±0.09 **B a** | 2.36 ±0.1 **A a** |
| For each parameter assessed means labelled with the same capital letter within the same row and same lower-case letter within the same column are not significant different (Tukey’s honestly significant difference test P<0.05) | | | |

| ***Table S14*.** Main effect means ± SE and *p*-values for the effects and interaction between wheat species, farming system and flour type on total concentration of phenolic acids detected by HPLC in flour collected from UK and DE between 2015 and 2016 (results are expressed on a flour dry weight basis) | | | | |
| --- | --- | --- | --- | --- |
|  | **Total phenolic components (HPLC)** | | | |
|  | **Free** | **Bound** | **Conjugated** | **Total** |
|  | µmol/g flour(DW) | | | |
| **Species** |  |  |  |  |
| Spelt (n=55) | 11.7 ±1.1 | 387 ±36 | 40.0 ±3.0 | 438 ±39 |
| Wheat (n=112) | 10.8 ±0.9 | 370 ±32 | 38.3 ±2.4 | 419 ±35 |
| **Farming system** |  |  |  |  |
| Conventional (n=84) | 9.6 ±0.9 | 351 ±37 | 33.7 ±2.4 | 394 ±40 |
| Organic (n=83) | 12.6 ±1.1 | 400 ±32 | 44.1 ±2.8 | 457 ±36 |
| **Flour Type** |  |  |  |  |
| White (n=90) | 6.5 ±0.6 | 122 ± 9 | 20.3 ±1.3 | 149 ±10 |
| Whole-grain (n=77) | 16.5 ±1.0 | 672 ±25 | 60.5 ±1.8 | 749 ±26 |
| **ANOVA** (p-values) |  |  |  |  |
| ***Main Effects*** |  |  |  |  |
| Species (SP) | NS | NS | NS | NS |
| Farming system (FS) | **0.0096** | NS | **<0.0001** | *0.0502* |
| Flour Type (FT) | **<0.0001** | **<0.0001** | **<0.0001** | **<0.0001** |
| ***Interactions*** |  |  |  |  |
| SP × FS | NS | NS | *0.0926* | NS |
| SP × FT | **0.0012 ^1^** | **0.0001** | **0.0036 ^1^** | **0.0001** |
| FS × FT | NS | NS | **0.0152 ^2^** | NS |
| SP × FS × FT | NS | **0.0066 ^3^** | NS | **0.0109 ^3^** |
| **^1^**See table S14.1 for Interaction means ± SE; **^2^**See table S14.2 for Interaction means ± SE; **^3^**See table S14.3 for Interaction means ± SE | | | | |

| ***Table S14.1*.** Interactions means ± SE for the effects of wheat species and flour type on total concentration of phenolic acids detected by HPLC in flour collected from UK and DE between 2015 and 2016 (results are expressed on a flour dry weight basis) | | | |
| --- | --- | --- | --- |
|  | **Factor 1** | **Factor 2** | |
|  |  | **flour type** | |
| **Fraction** | **wheat species** | White | Whole-grain |
|  |  | **Total phenolic components (HPCL)** | |
| **Free** | spelt | 7.9 ±1.2 **B a** | 14.6 ±1.5 **A a** |
| µmol/g flour (DW) | wheat | 6.0 ±0.7 **B a** | 17.7 ±1.4 **A a** |
|  |  |  | |
| **Conjugated** | spelt | 22 ±3 **B a** | 54 ±3 **A b** |
| µmol/g flour (DW) | wheat | 20 ±1 **B a** | 65 ±2 **A a** |
| For each parameter assessed means labelled with the same capital letter within the same row and same lower-case letter within the same column are not significant different (Tukey’s honestly significant difference test P<0.05) | | | |

| ***Table S14.2*.** Interactions means ± SE for the effects of farming system and flour type on total concentration of phenolic acids detected by HPLC in flour collected from UK and DE between 2015 and 2016 (results are expressed on a flour dry weight basis) | | | |
| --- | --- | --- | --- |
|  | **Factor 1** | **Factor 2** | |
|  |  | **flour Type** | |
| **Fraction** | **farming system** | White | Whole-grain |
|  |  | **Total phenolic components (HPCL)** | |
| **Conjugated** | conventional | 20 ±2 **B a** | 56 ±2 **A b** |
| µmol/g flour (DW) | organic | 21 ±2 **B a** | 64 ±3 **A a** |
| For each parameter assessed means labelled with the same capital letter within the same row and same lower-case letter within the same column are not significant different (Tukey’s honestly significant difference test P<0.05) | | | |

| ***Table S14.3*.** Interactions means ± SE for the effects of wheat species, flour type and farming system on total concentration of phenolic acids detected by HPCL in flour collected from UK and DE between 2015 and 2016 (results are expressed on a flour dry weight basis) | | | | |
| --- | --- | --- | --- | --- |
|  | **Factor 1** | **Factor 2** | **Factor 3** | |
|  |  |  | **flour Type** | |
| **Fraction** | **wheat species** | **farming system** | White | Whole-grain |
|  |  |  | **Total phenolic components (HPCL)** | |
| **Bound** | Spelt | conventional | 165 ±50 **B a** | 539 ±63 **A c** |
|  |  | organic | 104 ±17 **B a** | 606 ±27 **A bc** |
| µmol/g flour (DW) | Wheat | conventional | 113 ±10 **B a** | 781 ±56 **A a** |
|  |  | organic | 124 ±12 **B a** | 689 ±38 **A b** |
| **Total** | Spelt | conventional | 197 ±56 **B a** | 603 ±66 **A b** |
|  |  | organic | 132 ±20 **B a** | 677 ±29 **A b** |
| µmol/g flour (DW) | Wheat | conventional | 136 ±12 **B a** | 856 ±58 **A a** |
|  |  | organic | 153 ±15 **B a** | 779 ±41 **A a** |
| For each parameter assessed means labelled with the same capital letter within the same row and same lower-case letter within the same column are not significant different (Tukey’s honestly significant difference test P<0.05) | | | | |

## ***ANOVA 2: country × wheat species × farming system (for whole-grain flour only)***

| ***Table S 15*.**   Number (n) of common and spelt wheat **whole-grain** flour brands sampled and analysed for antioxidant activity, protein, phenolic and mineral micronutrient content in the retail survey in the UK and Germany in 2016. | | | | | | | |
| --- | --- | --- | --- | --- | --- | --- | --- |
|  |  | **Farming system** | | | | **Total** | |
| **Wheat Species** | **Country** | **Conventional** | | **Organic** | |  |  |
|  |  | **A&P** | **MIN** | **A&P** | **MIN** | **A&P** | **MIN** |
| **Wheat** | **UK** | 11 | 10** | 10 | 10* | **21** | **20** |
|  | **DE** | 3 | 3 | 6 | 6 | **9** | **9** |
| **TOTAL common wheat** | | **14** | **13** | **16** | **16** | **30** | **29** |
|  |  |  |  |  |  |  |  |
|  |  | **Farming system** | | | | **Total** | |
| **Wheat Species** | **Country** | **Conventional** | | **Organic** | |  |  |
|  |  | **A&P** | **MIN** | **A&P** | **MIN** | **A&P** | **MIN** |
| **Spelt** | **UK** | 5 | 5 | 7 | 7 | **12** | **12** |
|  | **DE** | 3 | 3 | 5 | 6 | **8** | **9** |
| **TOTAL spelt wheat** | | **8** | **8** | **12** | **13** | **20** | **21** |
|  |  |  |  |  |  |  |  |
| **TOTAL number of flour samples** | | ***22*** | ***21*** | ***28*** | ***29*** | ***50*** | ***50*** |
| *, includes one sample of self-rising flour; **, includes two samples of self-rising flour; data for Na, P, and Ca content obtained for self- rising flour were excluded from the statistical analyses of mineral composition data | | | | | | | |

| ***Table S16*.** Main effect means ± SE and *p*-values for the effects and interaction between country (UK and Germany), cereals species and farming system on phenolic content in flour collected in 2016 (results are expressed on a flour dry weight basis) | | | | |
| --- | --- | --- | --- | --- |
|  | **Phenolics Content** | | | |
|  | **Free** | **Bound** | **Conjugated** | **Total** |
| **Factor** | µmol GAE g^-1^ flour (DW) | | | |
| **Country** |  |  |  |  |
| DE (n=17) | 5.2 ±0.2 | 6.7 ±0.48 | 1.03 ±0.07 | 12.88 ±0.56 |
| UK (n=33) | 5.9 ±0.3 | 7.1 ±0.26 | 0.92 ±0.04 | 13.92 ±0.32 |
| **Species** |  |  |  |  |
| Spelt (n=20) | 5.3 ±0.2 | 6.2 ±0.36 | 0.88 ±0.06 | 12.35 ±0.38 |
| Wheat (n=30) | 5.9 ±0.3 | 7.5±0.28 | 1.00 ±0.04 | 14.37 ±0.34 |
| **Farming system** |  |  |  |  |
| Conventional (n=22) | 5.9 ±0.4 | 6.4 ±0.39 | 0.85 ±0.04 | 13.17 ±0.49 |
| Organic (n=28) | 5.4 ±0.2 | 7.4 ±0.27 | 1.04 ±0.05 | 13.87 ±0.34 |
| **ANOVA** (*p-*value) |  |  |  |  |
| ***Main Effects*** |  |  |  |  |
| Country (CT) | NS | NS | NS | NS |
| Species (SP) | NS | **0.0205** | *0.0713* | **0.0034** |
| Farming system (FS) | NS | **0.0111** | **0.0087** | *0.0792* |
| ***Interactions*** |  |  |  |  |
| **CT × SP** | NS | NS | NS | NS |
| **CT × FS** | NS | NS | NS | NS |
| **SP × FS** | NS | NS | NS | NS |
| **CT × SP × FS** | NS | NS | NS | NS |

| ***Table S17*.** Main effect means ± SE and *p*-values for the effects and interaction between country (UK and Germany), cereals species and farming system on Antioxidant activity by FRAP in flour collected in 2016 (results are expressed on a flour dry weight basis) | | | | |
| --- | --- | --- | --- | --- |
|  | **Antioxidant Activity by FRAP** | | | |
|  | **Free** | **Bound** | **Conjugated** | **Total** |
| **Factor** | µmol FeSO4.7H2O/g flour (DW) | | | |
| **Country** |  | | | |
| DE (n=17) | 1.7 ±0.1 | 5.5 ±0.4 | 0.93 ±0.08 | 8.2 ±0.6 |
| UK (n=33) | 2.0 ±0.1 | 6.0 ±0.2 | 0.83 ±0.04 | 8.9 ±0.3 |
| **Species** |  |  |  |  |
| Spelt (n=20) | 1.6 ±0.1 | 5.2 ±0.3 | 0.79 ±0.05 | 7.6 ±0.4 |
| Wheat (n=30) | 2.1 ±0.2 | 6.3 ±0.2 | 0.91 ±0.05 | 9.3 ±0.3 |
| **Farming system** |  |  |  |  |
| Conventional (n=22) | 1.6 ±0.2 | 5.4 ±0.3 | 0.71 ±0.03 | 7.7 ±0.4 |
| Organic (n=28) | 2.1 ±0.1 | 6.2 ±0.2 | 0.98 ±0.05 | 9.3 ±0.3 |
| ANOVA(p-values) |  |  |  |  |
| ***Main Effects*** |  |  |  |  |
| Country (CT) | NS | NS | NS | NS |
| Species (SP) | **0.0227** | **0.0217** | *0.0679* | **0.0045** |
| Farming system (FS) | **0.0112** | **0.0162** | **0.0003** | **0.0010** |
| ***Interactions*** |  |  |  |  |
| **CT × SP** | NS | NS | NS | NS |
| **CT × FS** | NS | NS | NS | NS |
| **SP × FS** | NS | NS | NS | NS |
| **CT × SP × FS** | NS | NS | NS | NS |

| ***Table S18*.** Main effect means ± SE and *p*-values for the effects and interaction between country (UK and Germany), cereals species and farming system on antioxidant activity by TEAC in flour collected in 2016 (results are expressed on a flour dry weight basis) | | | | |
| --- | --- | --- | --- | --- |
|  | **Antioxidant Activity by TEAC** | | | |
|  | **Free** | **Bound** | **Conjugated** | **Total** |
| **Factor** | µmol Trolox/g flour (DW) | | | |
| **Country** |  | | | |
| DE (n=17) | 2.24 ±0.09 | 9.11 ±0.5 | 1.3 ±0.22 | 12.7 ±0.7 |
| UK (n=33) | 2.31 ±0.07 | 9.89 ±0.3 | 0.9 ±0.08 | 13.1 ±0.3 |
| **Species** |  |  |  |  |
| Spelt (n=20) | 2.18 ±0.07 | 8.79 ±0.5 | 1.07 ±0.20 | 12.0 ±0.6 |
| Wheat (n=30) | 2.36 ±0.08 | 10.18 ±0.2 | 0.98 ±0.08 | 13.5 ±0.3 |
| **Farming system** |  |  |  |  |
| Conventional (n=22) | 2.12 ±0.08 | 9.23 ±0.5 | 0.83 ±0.09 | 12.2 ±0.6 |
| Organic (n=28) | 2.42 ±0.07 | 9.94 ±0.2 | 1.16 ±0.14 | 13.5 ±0.3 |
| **ANOVA**(p-values) |  |  |  |  |
| ***Main Effects*** |  |  |  |  |
| Country (CT) | NS | NS | *0.0999* | NS |
| Species (SP) | *0.0752* | **0.0180** | NS | **0.0330** |
| Farming system (FS) | **0.0026** | *0.0739* | **0.0261** | **0.0205** |
| ***Interactions*** |  |  |  |  |
| **CT × SP** | NS | NS | NS | NS |
| **CT × FS** | NS | NS | **0.0185 ^1^** | NS |
| **SP × FS** | NS | *0.0542* | NS | NS |
| **CT × SP × FS** | NS | NS | *0.0889* | NS |
| **^1^**See table S18.1 for Interaction means ± SE; | | | | |

| ***Table S18.1*.** Interactions means ± SE for the effects of country (UK and Germany) and production on total antioxidant activity of flour collected in 2016 by TEAC (results are expressed on a flour dry weight basis) | | | |
| --- | --- | --- | --- |
| **TEAC** | **Factor 1** | **Factor 2** | |
|  |  | **Farming system** | |
| **Parameter** | **Country** | Conventional | Organic |
| **Conjugated** | DE | 0.87 ±0.2 **B a** | 1.53 ±0.3 **A a** |
| µmol Trolox/g flour (DW) | UK | 0.82 ±0.1 **A a** | 0.92 ±0.1 **A b** |
| For each parameter assessed means labelled with the same capital letter within the same row and same lower-case letter within the same column are not significant different (Tukey’s honestly significant difference test P<0.05) | | | |

| ***Table S19*.** Main effect means ± SE and *p*-values for the effects and interaction between country (UK and Germany), cereals species and farming system on flavonoid content in flour collected in 2016 (results are expressed on a flour dry weight basis) | | | | |
| --- | --- | --- | --- | --- |
|  | **Flavonoid Content** | | | |
|  | **Free** | **Bound** | **Conjugated** | **Total** |
| **Factor** | µmol Catechin/g flour (DW) | | | |
| **Country** |  | | | |
| DE (n=17) | 0.19 ±0.01 | 0.95 ±0.07 | 0.12 ±0.01 | 1.26 ±0.08 |
| UK (n=33) | 0.21 ±0.02 | 0.98 ±0.05 | 0.18 ±0.06 | 1.37 ±0.08 |
| **Species** |  |  |  |  |
| Spelt (n=20) | 0.17 ±0.01 | 0.91 ±0.08 | 0.19 ±0.09 | 1.27 ±0.13 |
| Wheat (n=30) | 0.23 ±0.02 | 1.01 ±0.03 | 0.14 ±0.03 | 1.37 ±0.05 |
| **Farming system** |  |  |  |  |
| Conventional (n=22) | 0.19 ±0.02 | 0.90 ±0.06 | 0.09 ±0.01 | 1.18 ±0.06 |
| Organic (n=28) | 0.21 ±0.02 | 1.02 ±0.05 | 0.21 ±0.07 | 1.45 ±0.08 |
| **ANOVA**(p-values) |  |  |  |  |
| ***Main Effects*** |  |  |  |  |
| Country (CT) | NS | NS | NS | NS |
| Species (SP) | NS | NS | NS | NS |
| Farming system (FS) | NS | *0.0826* | **0.0443** | **0.0088** |
| ***Interactions*** |  |  |  |  |
| **CT × SP** | NS | NS | NS | NS |
| **CT × FS** | NS | NS | NS | NS |
| **SP × FS** | NS | NS | NS | NS |
| **CT × SP × FS** | NS | NS | NS | NS |

| ***Table S20*.** Main effect means ± SE and *p*-values for the effects and interaction between country (UK and Germany), cereals species and farming system on Ferulic acid content in flour collected in 2016 (results are expressed on a flour dry weight basis) | | | | |
| --- | --- | --- | --- | --- |
|  | **Ferulic Acid** | | | |
|  | **Free** | **Bound** | **Conjugated** | **Total** |
| **Factor** | µmol/g flour (DW) | | | |
| **Country** |  |  |  |  |
| DE (n=17) | 0.56 ±0.06 | 576 ±40 | 22 ±1 | 598 ±41 |
| UK (n=33) | 0.56 ±0.03 | 749 ±34 | 19 ±1 | 769 ±34 |
| **Species** |  |  |  |  |
| Spelt (n=20) | 0.45 ±0.04 | 574 ±36 | 18 ±1 | 592 ±37 |
| Wheat (n=30) | 0.64 ±0.03 | 767 ±34 | 21 ±1 | 789 ±34 |
| **Farming system** |  |  |  |  |
| Conventional (n=22) | 0.50 ±0.05 | 713 ±58 | 17 ±1 | 730 ±59 |
| Organic (n=28) | 0.61 ±0.03 | 672 ±22 | 22 ±1 | 695 ±23 |
| **ANOVA**(p-values) |  |  |  |  |
| ***Main Effects*** |  |  |  |  |
| Country (CT) | NS | **0.0160** | **0.0773** | **0.0177** |
| Species (SP) | **0.0047** | **0.0035** | **0.0064** | **0.0032** |
| Farming system (FS) | **0.0268** | NS | **0.0001** | NS |
| ***Interactions*** |  |  |  |  |
| **CT × SP** | NS | NS | NS | NS |
| **CT × FS** | NS | NS | NS | NS |
| **SP × FS** | NS | **0.0294 ^1^** | NS | **0.0326 ^1^** |
| **CT × SP × FS** | NS | NS | NS | NS |
| **^1^**See table S20.1 for Interaction means ± SE; | | | | |

| ***Table S20.1*.** Interactions means ± SE for the effects of species and farming system on phenolic acid concentration in flour collected in 2016 (results are expressed on a flour dry weight basis) | | | |
| --- | --- | --- | --- |
|  | **Factor 1** | **Factor 2** | |
|  |  | **Farming system** | |
| **Parameter** | **Species** | **Conventional** | **Organic** |
|  |  | **Ferulic Acid** | |
| **Bound** | Spelt | 500 ±80 **A b** | 624 ±24 **A a** |
| µmol/g flour (DW) | Wheat | 835 ±60 **A a** | 709 ±33 **B a** |
| **Total`** | Spelt | 515 ±81 **A b** | 644 ±24 **A a** |
| µmol/g flour (DW) | Wheat | 853 ±60 **A a** | 734 ±33 **B a** |
| For each parameter assessed means labelled with the same capital letter within the same row and same lower-case letter within the same column are not significant different (Tukey’s honestly significant difference test P<0.05) | | | |

| ***Table S21*.** Main effect means ± SE and *p*-values for the effects and interaction between country (UK and Germany), cereals species and farming system on macro nutrition in wholegrain flour collected in 2016 (results are expressed on a flour dry weight basis) | | | | | | | | |
| --- | --- | --- | --- | --- | --- | --- | --- | --- |
|  |  | **mineral macronutrients** | | | | | | |
|  | **Protein** | **N** | **Na*** | **P*** | **K** | **S** | **Ca*** | **Mg** |
|  | **%** | **mg/g** | **mg/kg** | **mg/g** | **mg/g** | **mg/g** | **mg/g** | **mg/g** |
| **Country** |  |  |  |  |  |  |  |  |
| DE (n=18) | 11.0 ±0.3 | 17.4 ±0.5 | 42 ±8 | 2.8 ±0.2 | 2.84 ±0.14 | 1.08 ±0.03 | 0.37 ±0.03 | 0.93 ±0.06 |
| UK (n=32) | 11.6 ±0.3 | 18.3 ±0.5 | 26 ±4 | 1.4 ±0.1 | 1.55 ±0.09 | 0.60 ±0.03 | 0.22 ±0.01 | 0.45 ±0.02 |
| **Species** |  |  |  |  |  |  |  |  |
| Spelt (n=21) | 11.7 ±0.3 | 18.5 ±0.5 | 23 ±1 | 2.1 ±0.2 | 1.99 ±0.18 | 0.83 ±0.07 | 0.25 ±0.02 | 0.64 ±0.07 |
| Wheat (n=29) | 11.2 ±0.3 | 17.7 ±0.5 | 39 ±7 | 1.8 ±0.2 | 2.03 ±0.15 | 0.74 ±0.04 | 0.30 ±0.03 | 0.61 ±0.05 |
| **Farming system** |  |  |  |  |  |  |  |  |
| Conventional (n=21) | 12.1 ±0.3 | 19.1 ±0.5 | 27 ±4 | 1.6 ±0.2 | 1.70 ±0.16 | 0.73 ±0.06 | 0.24 ±0.02 | 0.49 ±0.05 |
| Organic (n=29) | 10.8 ±0.3 | 17.1 ±0.5 | 36 ±6 | 2.2 ±0.2 | 2.24 ±0.15 | 0.81 ±0.05 | 0.30 ±0.02 | 0.72 ±0.05 |
| ANOVA(*p-*value) |  |  |  |  |  |  |  |  |
| ***Main Effects*** |  |  |  |  |  |  |  |  |
| Country (CT) | NS | NS | *0.0825* | **0.0002** | **0.0004** | **0.0001** | **0.0017** | **0.0002** |
| Species (SP) | NS | NS | **0.0343** | NS | NS | NS | **0.0176** | NS |
| Farming system (FS) | **0.0048** | **0.0048** | NS | **0.0106** | **0.0184** | NS | *0.0520* | **0.0021** |
| ***Interactions*** |  |  |  |  |  |  |  |  |
| **CT × SP** | *0.0999* | *0.0999* | NS | NS | NS | **0.0478 ^1^** | NS | NS |
| **CT × FS** | NS | NS | NS | NS | NS | *0.0888* | NS | NS |
| **SP × FS** | NS | NS | NS | NS | NS | NS | *0.0780* | NS |
| **CT × SP × FS** | NS | NS | NS | NS | NS | NS | NS | NS |
| **^1^**See table S22.1 for Interaction means ± SE;  *, excludes data of three self-rising flour sample. | | | | | | | | |

| ***Table S22*.** Main effect means ± SE and *p*-values for the effects and interaction between country (UK and Germany), cereals species and farming system on micro nutrition and toxic metals in wholegrain flour collected in 2016 (results are expressed on a flour dry weight basis) | | | | | | | | | |
| --- | --- | --- | --- | --- | --- | --- | --- | --- | --- |
|  | **Micro Nutrition** | | | | | |  | **Toxic metals** | |
|  | **Mn** | **Cu** | **Fe** | **Zn** | **Mo** | **Ni** |  | **Al** | **Cd** |
|  | **mg/kg** | **mg/kg** | **mg/kg** | **mg/kg** | **mg/kg** | **mg/kg** |  | **mg/kg** | **μg/kg** |
| **Country** |  |  |  |  |  |  |  |  |  |
| DE (n=18) | 24.5 ±1.8 | 7.9 ±0.4 | 34.0 ±2.2 | 25.2 ±1.8 | 0.55 ±0.06 | 0.57 ±0.09 |  | 5.4 ±0.4 | 55 ±3.6 |
| UK (n=32) | 12.9 ±0.8 | 4.3 ±0.3 | 18.5 ±1.5 | 12.4 ±0.7 | 0.30 ±0.03 | 0.28 ±0.07 |  | 2.7 ±0.5 | 31 ±3.4 |
| **Species** |  |  |  |  |  |  |  |  |  |
| Spelt (n=21) | 16.1 ±1.8 | 5.9 ±0.5 | 23.4 ±2.5 | 19.3 ±2.2 | 0.36 ±0.05 | 0.46 ±0.09 |  | 3.3 ±0.4 | 46 ±4.3 |
| Wheat (n=29) | 17.7 ±1.5 | 5.3 ±0.5 | 24.6 ±2.2 | 15.4 ±1.2 | 0.41 ±0.05 | 0.33 ±0.07 |  | 4.0 ±0.6 | 35 ±4.0 |
| **Farming system** |  |  |  |  |  |  |  |  |  |
| Conventional (n=21) | 13.3 ±1.4 | 4.5 ±0.5 | 20.2 ±2.3 | 12.4 ±1.3 | 0.22 ±0.01 | 0.27 ±0.04 |  | 2.4 ±0.4 | 37 ±4.1 |
| Organic (n=29) | 19.8 ±1.5 | 6.4 ±0.4 | 26.9 ±2.2 | 20.4 ±1.6 | 0.51 ±0.05 | 0.47 ±0.09 |  | 4.6 ±0.6 | 42 ±4.3 |
| ANOVA(*p-*value) |  |  |  |  |  |  |  |  |  |
| ***Main Effects*** |  |  |  |  |  |  |  |  |  |
| Country (CT) | **0.0009** | **0.0005** | **0.0014** | **0.0002** | **0.0020** | **0.0589** |  | **0.0094** | **0.0117** |
| Species (SP) | *0.0764* | NS | NS | *0.0692* | *0.0621* | NS |  | NS | NS |
| Farming system (FS) | **0.0020** | **0.0055** | **0.0482** | **0.0001** | **<0.0001** | **0.1127** |  | **0.0059** | NS |
| ***Interactions*** |  |  |  |  |  |  |  |  |  |
| **CT × SP** | NS | NS | NS | **0.0208 ^1^** | NS | **0.0385 ^1^** |  | NS | NS |
| **CT × FS** | NS | NS | NS | NS | **0.0060 ^2^** | NS |  | NS | NS |
| **SP × FS** | NS | NS | NS | NS | **0.0242 ^2^** | NS |  | **0.0386 ^2^** | NS |
| **CT × SP × FS** | NS | NS | *0.0951* | NS | NS | NS |  | NS | NS |
| **^1^**See table S22.1 for Interaction means ± SE; **^2^**See table S22.2 for Interaction means ± SE; **^3^**See table S20.3 for Interaction means ± SE; | | | | | | | | | |

| ***Table S22.1*** Interactions means ± SE for the effects of country (UK and Germany) and species on S and Zn content in flour collected in 2016 (results are expressed on a flour dry weight basis) | | | |
| --- | --- | --- | --- |
|  | **Factor 1** | **Factor 2** | |
|  |  | **Species** | |
| **Parameter** | **Country** | spelt | wheat |
| **S** | DE | 1.15 ±0.05 **A a** | 1.01 ±0.01 **B a** |
| mg/g(DW) | UK | 0.59 ±0.02 **A b** | 0.61 ±0.04 **A b** |
| **Zn** | DE | 28.4 ±3.0 **A a** | 22.0 ±1.6 **B a** |
| mg/kg (DW) | UK | 12.5 ±0.7 **A b** | 12.4 ±1.1 **A b** |
| For each parameter assessed means labelled with the same capital letter within the same row and same lower-case letter within the same column are not significant different (Tukey’s honestly significant difference test P<0.05) | | | |

| ***Table S22.2*** Interactions means ± SE for the effects of country (UK and Germany) and species on Mo content in flour collected in 2016 (results are expressed on a flour dry weight basis) | | | |
| --- | --- | --- | --- |
|  | **Factor 1** | **Factor 2** | |
|  |  | **Farming system** | |
| **Parameter** | **Country** | Conventional | Organic |
| **Mo** | DE | 0.24 ±0.02 **B a** | 0.70 ±0.06 **A a** |
| mg/kg (DW) | UK | 0.21 ±0.02 **B a** | 0.38 ±0.04 **A b** |
| For each parameter assessed means labelled with the same capital letter within the same row and same lower-case letter within the same column are not significant different (Tukey’s honestly significant difference test P<0.05) | | | |

| ***Table S22.3*** Interactions means ± SE for the effects of species and farming systems on Mo and Al content in flour collected from UK and Germany in 2016 (results are expressed on a flour dry weight basis) | | | |
| --- | --- | --- | --- |
|  | **Factor 1** | **Factor 2** | |
|  |  | **Farming system** | |
| **Parameter** | **Species** | Conventional | Organic |
| **Mo** | Spelt | 0.24 ±0.03 **B a** | 0.43 ±0.06 **A b** |
| mg/g(DW) | Wheat | 0.20 ±0.01 **B a** | 0.58 ±0.06 **A a** |
| **Al** | Spelt | 3.01 ±0.69 **A a** | 3.52 ±0.56 **A b** |
| mg/kg (DW) | Wheat | 2.02 ±0.38 **B a** | 5.51 ±0.89 **A a** |
| For each parameter assessed means labelled with the same capital letter within the same row and same lower-case letter within the same column are not significant different (Tukey’s honestly significant difference test P<0.05) | | | |

## ***ANOVA 3: year (2015, 2016) × wheat species × farming system (for UK whole-grain flour only)***

| ***Table S 23.***  Number (n) of wheat flour brands sampled and analysed for antioxidant activity and phenolic concentration (A&P) and mineral content (MIN) in the retail survey in UK between 2015 and 2016. | | | | | | | |
| --- | --- | --- | --- | --- | --- | --- | --- |
|  |  | **Year** | | | | **TOTAL** | |
| **Wheat Species** | **Farming System** | **2015** | | **2016** | |  |  |
|  |  | **A&P** | **MIN** | **A&P** | **MIN** | **A&P** | **MIN** |
| **Common wheat** | **Conventional** | 8 | 8 | 11 | 10** | **19** | **18** |
|  | **Organic** | 8 | 7 | 10 | 10* | **18** | **17** |
| **TOTAL common wheat** | | **16** | **15** | **21** | **20** | **37** | **35** |
|  |  |  |  |  |  |  |  |
|  |  | **Year** | | | | **TOTAL** | |
| **Wheat Species** | **Farming System** | **2015** | | **2016** | |  |  |
|  |  | **A&P** | **MIN** | **A&P** | **MIN** | **A&P** | **MIN** |
| **Spelt wheat** | **Conventional** | 1 | 1 | 5 | 5 | **6** | **6** |
|  | **Organic** | 6 | 6 | 7 | 7 | **13** | **13** |
| **TOTAL spelt wheat** | | **7** | **7** | **12** | **12** | **19** | **19** |
|  |  |  |  |  |  |  |  |
| **TOTAL number of flour samples** | | ***23*** | ***22*** | ***33*** | ***32*** | ***56*** | ***54*** |
| *, includes one sample of self-rising flour; **, includes two samples of self-rising flour; data for Na, P, and Ca content obtained for self- rising flour were excluded from the statistical analyses of mineral composition data | | | | | | | |

| ***Table S24.*** Main effect means ± SE and *p*-values for the effects and interaction between year (2015 and 2016), cereals species and farming system on phenolic content in UK whole-grain flour collected between 2015 and 2016 (results are expressed on a flour dry weight basis) | | | | |
| --- | --- | --- | --- | --- |
|  | **Phenolics Content** | | | |
|  | **Free** | **Bound** | **Conjugated** | **Total** |
| **Factor** | µmol GAE g^-1^ flour (DW) | | | |
| **Year** |  |  |  |  |
| 2015 (n=23) | 3.5 ±0.1 | 5.0 ±0.2 | 0.80 ±0.05 | 9.4 ±0.2 |
| 2016 (n=33) | 5.9 ±0.3 | 7.1 ±0.3 | 0.92 ±0.04 | 13.9 ±0.3 |
| **Species** |  |  |  |  |
| Spelt (n=19) | 4.7 ±0.2 | 5.9 ±0.3 | 0.81 ±0.05 | 11.4 ±0.5 |
| Wheat (n=37) | 5.0 ±0.3 | 6.5 ±0.3 | 0.90 ±0.04 | 12.4 ±0.5 |
| **Farming system** |  |  |  |  |
| Conventional (n=25) | 5.4 ±0.4 | 6.0 ±0.3 | 0.80 ±0.04 | 12.2 ±0.6 |
| Organic (n=31) | 4.5 ±0.2 | 6.5 ±0.3 | 0.93 ±0.04 | 11.9 ±0.5 |
| **ANOVA**(p-values) |  |  |  |  |
| ***Main Effects*** |  |  |  |  |
| Year (YR) | **0.0001** | **0.0005** | *0.0749* | **<0.0001** |
| Species (SP) | NS | *0.0577* | NS | **0.0074** |
| Farming system (FS) | *0.0796* | **0.0165** | **0.0084** | NS |
| ***Interactions*** |  |  |  |  |
| **YR × SP** | NS | NS | NS | *0.0594* |
| **YR × FS** | NS | NS | NS | NS |
| **SP × FS** | NS | NS | NS | NS |
| **YR × SP × FS** | NS | NS | NS | NS |

| ***Table S25*.** Main effect means ± SE and *p*-values for the effects and interaction year, cereals species and farming system on antioxidant activity by FRAP of UK whole-grain flour collected between 2015 and 2016 (results are expressed on a flour dry weight basis) | | | | |
| --- | --- | --- | --- | --- |
|  | **Antioxidant Activity by FRAP** | | | |
|  | **Free** | **Bound** | **Conjugated** | **Total** |
| **Factor** | µmol FeSO4.7H2O/g flour (DW) | | | |
| **Year** |  | | | |
| 2015 (n=23) | 1.3 ±0.1 | 5.4 ±0.2 | 1.14 ±0.28 | 7.8 ±0.4 |
| 2016 (n=33) | 2.0 ±0.1 | 6.0 ±0.2 | 0.83 ±0.04 | 8.9 ±0.3 |
| **Species** |  |  |  |  |
| Spelt (n=19) | 1.5 ±0.1 | 5.4 ±0.3 | 0.74 ±0.04 | 7.6 ±0.3 |
| Wheat (n=37) | 1.8 ±0.1 | 6.0 ±0.2 | 1.07 ±0.17 | 8.9 ±0.3 |
| **Farming system** |  |  |  |  |
| Conventional (n=25) | 1.6 ±0.1 | 5.5 ±0.2 | 1.06 ±0.26 | 8.2 ±0.4 |
| Organic (n=31) | 1.8 ±0.1 | 6.0 ±0.2 | 0.88 ±0.05 | 8.7 ±0.3 |
| **ANOVA**(p-values) |  |  |  |  |
| ***Main Effects*** |  |  |  |  |
| Year (YR) | **0.0029** | *0.0577* | NS | **0.0310** |
| Species (SP) | **0.0229** | *0.0711* | NS | **0.0071** |
| Farming system (FS) | **0.0410** | **0.0241** | NS | **0.0341** |
| ***Interactions*** |  |  |  |  |
| **YR × SP** | NS | NS | NS | NS |
| **YR × FS** | NS | NS | **0.0470 ^1^** | **0.0257 ^1^** |
| **SP × FS** | NS | NS | NS | NS |
| **YR × SP × FS** | NS | NS | NS | NS |
| **^1^**See table S25.1 for Interaction means ± SE; | | | | |

| ***Table S25.1*.** Interactions means ± SE for the effects of year and farming system on total antioxidant activity by FRAP of UK whole-grain flour collected between 2015 and 2016 (results are expressed on a flour dry weight basis) | | | |
| --- | --- | --- | --- |
| **FRAP** | **Factor 1** | **Factor 2** | |
|  |  | **Flour type** | |
| **Parameter** | **Year** | Conventional | Organic |
| **Conjugated** | 2015 | 1.68 ±0.68 **A a** | 0.80 ±0.07 **B a** |
| µmol FeSO4.7H2O/g flour (DW) | 2016 | 0.71 ±0.03 **A b** | 0.95 ±0.06 **A a** |
| **Total** | 2015 | 8.20 ±0.74 **A a** | 7.56 ±0.38 **A b** |
| µmol FeSO4.7H2O/g flour (DW) | 2016 | 8.12 ±0.39 **B a** | 9.59 ±0.33 **A a** |
| For each parameter assessed means labelled with the same capital letter within the same row and same lower-case letter within the same column are not significant different (Tukey’s honestly significant difference test P<0.05) | | | |

| ***Table S26*.** Main effect means ± SE and *p*-values for the effects and interaction between year, cereals species and farming system on antioxidant activity by TEAC of UK whole-grain flour collected between 2015 and 2016 (results are expressed on a flour dry weight basis) | | | | |
| --- | --- | --- | --- | --- |
|  | **Antioxidant Activity by TEAC** | | | |
|  | **Free** | **Bound** | **Conjugated** | **Total** |
| **Factor** | µmol Trolox/g flour (DW) | | | |
| **Year** |  | | | |
| 2015 (n=23) | 2.3 ±0.1 | 12.6 ±0.7 | 3.5 ±0.8 | 18.4 ±0.8 |
| 2016 (n=33) | 2.3 ±0.1 | 9.9 ±0.3 | 0.9 ±0.1 | 13.1 ±0.3 |
| **Species** |  |  |  |  |
| Spelt (n=19) | 2.1 ±0.1 | 10.3 ±0.7 | 1.5 ±0.4 | 13.9 ±0.8 |
| Wheat (n=37) | 2.4 ±0.1 | 11.4 ±0.4 | 2.2 ±0.5 | 16.0 ±0.7 |
| **Farming system** |  |  |  |  |
| Conventional (n=25) | 2.2 ±0.1 | 10.5 ±0.6 | 1.6 ±0.4 | 14.3 ±0.7 |
| Organic (n=31) | 2.3 ±0.1 | 11.4 ±0.5 | 2.3 ±0.6 | 16.1 ±0.8 |
| **ANOVA**(p-values) |  |  |  |  |
| **Main Effects** |  |  |  |  |
| Year (YR) | NS | **0.0083** | **0.0162** | **0.0002** |
| Species (SP) | **0.0097** | NS | NS | **0.0495** |
| Farming system (FS) | **0.0294** | NS | NS | **0.0343** |
| ***Interactions*** |  |  |  |  |
| **YR × SP** | *0.0749* | NS | NS | NS |
| **YR × FS** | *0.0528* | NS | NS | NS |
| **SP × FS** | NS | NS | NS | NS |
| **YR × SP × FS** | *0.0484* | **0.0309 ^1^** | NS | NS |
| **^1^**See table S26.1 for Interaction means ± SE; | | | | |

| ***Table S26.1*.** Interactions means ± SE for the effects of species, farming system and flour type on total antioxidant activity by TEAC of UK whole-grain flour collected between 2015 and 2016 (results are expressed on a flour dry weight basis) | | | | |
| --- | --- | --- | --- | --- |
|  | **Factor 1** | **Factor 2** | **Factor 3** | |
|  |  |  | **Farming system** | |
| **Parameter** | **Year** | **Species** | Conventional | Organic |
| **TEAC (Total)**  µmol Trolox/g flour (DW) | 2015 | Spelt | 15.3 ±0.0 **A a** | 12.0 ±1.3 **A a** |
|  |  | Wheat | 11.7 ±1.2 **A ab** | 13.6 ±1.2 **A a** |
|  | 2016 | Spelt | 7.9 ±1.2 **A c** | 9.7 ±0.3 **A a** |
|  |  | Wheat | 10.3 ±0.4 **A b** | 10.6 ±0.3 **A a** |
| For each parameter assessed means labelled with the same capital letter within the same row and same lower-case letter within the same column are not significant different (Tukey’s honestly significant difference test P<0.05) | | | | |

| ***Table S27*.** Main effect means ± SE and *p*-values for the effects and interaction between year, cereals species and farming system on flavonoid content in UK whole-grain flour collected between 2015 and 2016 (results are expressed on a flour dry weight basis) | | | | |
| --- | --- | --- | --- | --- |
|  | **Flavonoid Content** | | | |
|  | **Free** | **Bound** | **Conjugated** | **Total** |
| **Factor** | µmol Catechin/g flour (DW) | | | |
| **Year** |  | | | |
| 2015 (n=23) | 0.24 ±0.02 | 1.26 ±0.19 | 0.14 ±0.01 | 1.6 ±0.2 |
| 2016 (n=33) | 0.21 ±0.02 | 0.98 ±0.05 | 0.18 ±0.06 | 1.4 ±0.1 |
| **Species** |  |  |  |  |
| Spelt (n=19) | 0.20 ±0.02 | 1.03 ±0.09 | 0.21 ±0.09 | 1.4 ±0.1 |
| Wheat (n=37) | 0.24 ±0.02 | 1.13 ±0.12 | 0.14 ±0.02 | 1.5 ±0.1 |
| **Farming system** |  |  |  |  |
| Conventional (n=25) | 0.21 ±0.02 | 1.12 ±0.18 | 0.09 ±0.01 | 1.4 ±0.2 |
| Organic (n=31) | 0.24 ±0.02 | 1.07 ±0.05 | 0.22 ±0.06 | 1.5 ±0.1 |
| **ANOVA**(p-values) |  |  |  |  |
| ***Main Effects*** |  |  |  |  |
| Year (YR) | NS | NS | NS | NS |
| Species (SP) | NS | NS | NS | NS |
| Farming system (FS) | NS | NS | **0.0339** | NS |
| ***Interactions*** |  |  |  |  |
| **YR × SP** | NS | NS | NS | NS |
| **YR × FS** | NS | NS | NS | *0.0791* |
| **SP × FS** | NS | NS | NS | NS |
| **YR × SP × FS** | NS | NS | NS | NS |

| ***Table S28*.** Main effect means ± SE and *p*-values for the effects and interaction between year, cereals species and farming system on Ferulic acid content in UK whole-grain flour collected between 2015 and 2016 (results are expressed on a flour dry weight basis) | | | | |
| --- | --- | --- | --- | --- |
|  | **Ferulic Acid** | | | |
|  | **Free** | **Bound** | **Conjugated** | **Total** |
| **Factor** | µmol/g flour (DW) | | | |
| **Year** |  |  |  |  |
| 2015 (n=23) | 2.73 ±0.17 | 473 ±20 | 18.9 ±1.1 | 495 ±21 |
| 2016 (n=33) | 0.56 ±0.03 | 749 ±34 | 18.9 ±0.7 | 769 ±34 |
| **Species** |  |  |  |  |
| Spelt (n=19) | 1.07 ±0.21 | 550 ±31 | 17.6 ±0.9 | 569 ±31 |
| Wheat (n=37) | 1.65 ±0.21 | 680 ±38 | 19.5 ±0.8 | 701 ±38 |
| **Farming system** |  |  |  |  |
| Conventional (n=25) | 1.31 ±0.25 | 685 ±50 | 16.9 ±0.8 | 704 ±50 |
| Organic (n=31) | 1.56 ±0.21 | 596 ±29 | 20.5 ±0.8 | 618 ±30 |
| **ANOVA**(p-values) |  |  |  |  |
| ***Main Effects*** |  |  |  |  |
| Year (YR) | **<0.0001** | **0.0002** | 0.9842 | **0.0002** |
| Species (SP) | **0.0072** | **0.0029** | 0.1133 | **0.0029** |
| Farming system (FS) | 0.3039 | 0.2777 | **0.001** | 0.3339 |
| ***Interactions*** |  |  |  |  |
| **YR × SP** | **0.0317 ^1^** | **0.0364 ^1^** | *0.0502* | **0.0344 ^1^** |
| **YR × FS** | NS | NS | NS | NS |
| **SP × FS** | NS | **0.0332 ^2^** | NS | **0.0385 ^2^** |
| **YR × SP × FS** | NS | NS | NS | NS |
| **^1^**See table S28.1 for Interaction means ± SE; **^2^**See table S28.2 for Interaction means ± SE; | | | | |

| ***Table S28.1*.** Interactions means ± SE for the effects of year and species on phenolic acid concentration in UK whole-grain flour collected between 2015 and 2016 (results are expressed on a flour dry weight basis) | | | |
| --- | --- | --- | --- |
|  | **Factor 1** | **Factor 2** | |
|  |  | **Species** | |
| **Parameter** | **Year** | **Spelt** | **Wheat** |
|  |  | **Ferulic Acid** | |
| **Free** | 2015 | 2.17 ±0.15 **B a** | 2.98 ±0.21 **A a** |
| µmol/g flour (DW) | 2016 | 0.43 ±0.06 **A b** | 0.63 ±0.03 **A b** |
| **Bound** | 2015 | 448 ±13.6 **A b** | 484 ±29 **A b** |
| µmol/g flour (DW) | 2016 | 610 ±39.7 **B a** | 829 ±38 **A a** |
| **Total** | 2015 | 470 ±14.1 **A b** | 506 ±29 **A b** |
| µmol/g flour (DW) | 2016 | 627 ±40.3 **B a** | 850 ±38 **A a** |
| For each parameter assessed means labelled with the same capital letter within the same row and same lower-case letter within the same column are not significant different (Tukey’s honestly significant difference test P<0.05) | | | |

| ***Table S28.2*.** Interactions means ± SE for the effects of species and flour type on phenolic acid concentration in UK whole-grain flour collected between 2015 and 2016 (results are expressed on a flour dry weight basis) | | | |
| --- | --- | --- | --- |
|  | **Factor 1** | **Factor 2** | |
|  |  | **Farming system** | |
| **Parameter** | **Species** | **Conventional** | **Organic** |
|  |  | **Ferulic Acid** | |
| **Bound** | Spelt | 540 ±71 **A b** | 555 ±34 **A a** |
| µmol/g flour (DW) | Wheat | 731 ±59 **A a** | 625 ±44 **B a** |
| **Total** | Spelt | 556 ±71 **A b** | 575 ±34 **A a** |
| µmol/g flour (DW) | Wheat | 750 ±59 **A a** | 649 ±44 **B a** |
| For each parameter assessed means labelled with the same capital letter within the same row and same lower-case letter within the same column are not significant different (Tukey’s honestly significant difference test P<0.05) | | | |

| ***Table S29*.** Main effect means ± SE and *p*-values for the effects and interaction between year, cereals species and farming system on Sinapic acids content in UK whole-grain flour collected between 2015 and 2016 (results are expressed on a flour dry weight basis) | | | | |
| --- | --- | --- | --- | --- |
|  | **Sinapic Acid** | | | |
|  | **Free** | **Bound** | **Conjugated** | **Total** |
| **Factor** | µmol/g flour (DW) | | | |
| **Year** |  |  |  |  |
| 2015 (n=23) | 0.19 ±0.02 | 14 ±1.1 | 21 ±1.6 | 36 ±2.3 |
| 2016 (n=33) | 10.93 ±0.61 | 22 ±1.5 | 26 ±1.4 | 59 ±2.7 |
| **Species** |  |  |  |  |
| Spelt (n=19) | 6.62 ±1.27 | 13 ±1.1 | 19 ±1.4 | 39 ±2.9 |
| Wheat (n=37) | 6.47 ±1.03 | 22 ±1.4 | 26 ±1.3 | 55 ±3.0 |
| **Farming system** |  |  |  |  |
| Conventional (n=25) | 6.37 ±1.08 | 21 ±1.9 | 23 ±1.3 | 51 ±3.5 |
| Organic (n=31) | 6.65 ±1.16 | 17 ±1.2 | 24 ±1.7 | 48 ±3.3 |
| **ANOVA**(p-values) |  |  |  |  |
| ***Main Effects*** |  |  |  |  |
| Year (YR) | **<.0001** | **0.0009** | *0.0742* | **0.0001** |
| Species (SP) | NS | **0.0001** | **0.0025** | **0.0002** |
| Farming system (FS) | *0.0622* | NS | *0.0845* | NS |
| ***Interactions*** |  |  |  |  |
| **YR × SP** | NS | NS | NS | NS |
| **YR × FS** | NS | NS | NS | NS |
| **SP × FS** | NS | *0.0926* | NS | NS |
| **YR × SP × FS** | NS | **0.0285 ^1^** | NS | NS |
| **^1^**See table S29.1 for Interaction means ± SE; | | | | |

| ***Table S29.1*.** Interactions means ± SE for the effects of year, species and farming system on phenolic acid concentration in UK whole-grain flour collected between 2015 and 2016 (results are expressed on a flour dry weight basis) | | | | |
| --- | --- | --- | --- | --- |
|  | **Factor 1** | **Factor 2** | **Factor 3** | |
|  |  |  | **Farming system** | |
| **Parameter** | **Year** | **Species** | **Conventional** | **Organic** |
|  |  |  | **Sinap Acid** | |
| **Bound**  µmol/g flour (DW) | 2015 | Spelt | 15.14 ±0.00 **A b** | 9.03 ±0.71 **A b** |
|  |  | Wheat | 15.14 ±1.39 **A b** | 16.80 ±2.37 **A c** |
|  | 2016 | Spelt | 13.55 ±2.39 **A b** | 16.13 ±1.54 **A c** |
|  |  | Wheat | 29.92 ±2.17 **A a** | 22.13 ±1.60 **B a** |
| For each parameter assessed means labelled with the same capital letter within the same row and same lower-case letter within the same column are not significant different (Tukey’s honestly significant difference test P<0.05) | | | | |

| ***Table S30*.** Main effect means ± SE and *p*-values for the effects and interaction between year, cereals species and farming system on macro nutrient concentrations in UK whole-grain flour collected between 2015 and 2016 (results are expressed on a flour dry weight basis) | | | | | | | | |
| --- | --- | --- | --- | --- | --- | --- | --- | --- |
|  | **Macro Nutrient** | | | | | | | |
|  | **Protein** | **N** | **Na*** | **P*** | **K** | **S** | **Ca*** | **Mg** |
|  | **%** | **mg/g** | **mg/kg** | **mg/g** | **mg/g** | **mg/g** | **mg/g** | **mg/g** |
| **Year** |  |  |  |  |  |  |  |  |
| 2015 (n=22) | 11.57 ±0.3 | 18.3 ±0.5 | 59 ±4 | 2.5 ±0.1 | 2.5 ±0.1 | 1.09 ±0.03 | 0.32 ±0.01 | 0.80 ±0.03 |
| 2016 (n=32) | 11.56 ±0.3 | 18.3 ±0.5 | 26 ±4 | 1.4 ±0.1 | 1.6 ±0.1 | 0.60 ±0.03 | 0.22 ±0.01 | 0.45 ±0.02 |
| **Species** |  |  |  |  |  |  |  |  |
| Spelt (n=19) | 11.49 ±0.3 | 18.2 ±0.5 | 26 ±3 | 2.1 ±0.2 | 1.9 ±0.2 | 0.81 ±0.07 | 0.24 ±0.01 | 0.61 ±0.06 |
| Wheat (n=35) | 11.60 ±0.3 | 18.4 ±0.5 | 49 ±5 | 1.7 ±0.1 | 1.9 ±0.1 | 0.80 ±0.05 | 0.27 ±0.01 | 0.58 ±0.03 |
| **Farming system** |  |  |  |  |  |  |  |  |
| Conventional (n=24) | 12.08 ±0.3 | 19.2 ±0.5 | 42 ±6 | 1.6 ±0.1 | 1.8 ±0.1 | 0.75 ±0.05 | 0.24 ±0.01 | 0.51 ±0.04 |
| Organic (n=30) | 11.15 ±0.3 | 17.7 ±0.5 | 39 ±4 | 2.0 ±0.1 | 2.1 ±0.1 | 0.84 ±0.05 | 0.27 ±0.01 | 0.65 ±0.04 |
| ANOVA(*p-*value) |  |  |  |  |  |  |  |  |
| ***Main Effects*** |  |  |  |  |  |  |  |  |
| Year (YR) | NS | NS | **0.0002** | **<0.0001** | **0.0001** | **<0.0001** | **0.0003** | **<0.0001** |
| Species (SP) | NS | NS | **0.0013** | **0.0009** | NS | NS | NS | 0.1302 |
| Farming system (FS) | **0.0179** | **0.0179** | NS | **0.0061** | **0.0326** | NS | **0.0202** | **0.0020** |
| ***Interactions*** |  |  |  |  |  |  |  |  |
| **YR × SP** | NS | NS | *0.0569* | **0.0071** | *0.0834* | *0.0677* | NS | **0.0395 ^1^** |
| **YR × FS** | NS | NS | **0.0122 ^2^** | NS | *0.0607* | NS | NS | NS |
| **SP × FS** | NS | NS | NS | NS | NS | NS | NS | NS |
| **YR × SP × FS** | NS | NS | *0.0830* | **0.0110 ^3^** | **0.0401 ^3^** | NS | NS | **0.0194 ^3^** |
| **^1^**See table S30.1 for Interaction means ± SE; **^2^**See table S30.2 for Interaction means ± SE; **^3^**See table S30.3 for Interaction means ± SE;  *, excludes data of three self-rising flour samples | | | | | | | | |

| ***Table S30.1*.** Interactions means ± SE for the effects of year and species on Mg content in UK whole-grain flour collected between 2015 and 2016 (results are expressed on a flour dry weight basis) | | | |
| --- | --- | --- | --- |
|  | **Factor 1** | **Factor 2** | |
|  |  | **Species** | |
| **Parameter** | **Year** | spelt | Wheat |
| **Mg** | 2015 | 0.91 ±0.04 **A a** | 0.75 ±0.03 **B a** |
| mg/g(DW) | 2016 | 0.43 ±0.03 **A b** | 0.46 ±0.03 **A b** |
| For each parameter assessed means labelled with the same capital letter within the same row and same lower-case letter within the same column are not significant different (Tukey’s honestly significant difference test P<0.05) | | | |

| ***Table S30.2*.** Interactions means ± SE for the effects of year and farming system on Na content in UK whole-grain flour collected between 2015 and 2016 (results are expressed on a flour dry weight basis) | | | |
| --- | --- | --- | --- |
|  | **Factor 1** | **Factor 2** | |
|  |  | **Farming system** | |
| **Parameter** | **Year** | Conventional | Organic |
| **Na** | 2015 | 72.6 ±5.2 **A a** | 49.0 ±4.4 **B a** |
| **mg/kg** | 2016 | 20.1 ±0.6 **A b** | 31.0 ±6.7 **A b** |
| For each parameter assessed means labelled with the same capital letter within the same row and same lower-case letter within the same column are not significant different (Tukey’s honestly significant difference test P<0.05) | | | |

| ***Table S30.3*.** Interactions means ± SE for the effects of species, farming system and flour type on K and Mg content in UK whole-grain flour collected between 2015 and 2016 (results are expressed on a flour dry weight basis) | | | | |
| --- | --- | --- | --- | --- |
|  | **Factor 1** | **Factor 2** | **Factor 3** | |
|  |  |  | **Farming system** | |
| **Parameter** | **Year** | **Species** | Conventional | Organic |
| **K**  mg/g(DW) | 2015 | Spelt | 2.20 ±0.00 **A a** | 2.76 ±0.05 **A a** |
|  |  | Wheat | 2.46 ±0.08 **A a** | 2.24 ±0.13 **A b** |
|  | 2016 | Spelt | 1.36 ±0.15 **A b** | 1.46 ±0.06 **A d** |
|  |  | Wheat | 1.34 ±0.05 **B b** | 1.91 ±0.24 **A b** |
| **Mg**  mg/g(DW) | 2015 | Spelt | 0.69 ±0.00 **B a** | 0.95 ±0.01 **A a** |
|  |  | Wheat | 0.75 ±0.05 **A a** | 0.74 ±0.05 **A b** |
|  | 2016 | Spelt | 0.39 ±0.06 **A b** | 0.46 ±0.03 **A c** |
|  |  | Wheat | 0.3 7±0.03 **B b** | 0.55 ±0.05 **A c** |
| **P**  mg/g(DW) | 2015 | Spelt | 2.30 ±0.00 **B a** | 3.22 ±0.59 **A a** |
|  |  | Wheat | 2.22 ±0.16 **A a** | 2.20 ±0.12 **A b** |
|  | 2016 | Spelt | 1.38 ±0.16 **A b** | 1.51 ±0.08 **A c** |
|  |  | Wheat | 1.09 ±0.07 **B b** | 1.52 ±0.15 **A c** |
| For each parameter assessed means labelled with the same capital letter within the same row and same lower-case letter within the same column are not significant different (Tukey’s honestly significant difference test P<0.05) | | | | |

| ***Table S31*.** Main effect means ± SE and *p*-values for the effects and interaction between year, cereals species and farming system on micronutrient and toxic metal concentrations in UK whole-grain flour collected between 2015 and 2016 (results are expressed on a flour dry weight basis) | | | | | | | | | |
| --- | --- | --- | --- | --- | --- | --- | --- | --- | --- |
|  | **Micro Nutrient** | | | | | |  | **Toxic metals** | |
|  | **Mn** | **Cu** | **Fe** | **Zn** | **Mo** | **Ni** |  | **Al** | **Cd** |
|  | **mg/kg** | **mg/kg** | **mg/kg** | **mg/kg** | **mg/kg** | **mg/kg** |  | **mg/kg** | **μg/kg** |
| **Year** |  |  |  |  |  |  |  |  |  |
| 2015 (n=22) | 25 ±1.4 | 5.6 ±0.7 | 30 ±1 | 21.7 ±1.1 | 0.53 ±0.05 | 0.33 ±0.04 |  | 5.1 ±0.56 | 47 ±2.9 |
| 2016 (n=32) | 13 ±0.8 | 4.3 ±0.3 | 19 ±2 | 12.4 ±0.7 | 0.30 ±0.03 | 0.28 ±0.07 |  | 2.7 ±0.51 | 31 ±3.4 |
| **Species** |  |  |  |  |  |  |  |  |  |
| Spelt (n=19) | 17 ±2.2 | 5.6 ±0.8 | 22 ±2 | 17.9 ±1.8 | 0.41 ±0.07 | 0.33 ±0.06 |  | 3.1 ±0.55 | 44 ±4.4 |
| Wheat (n=35) | 18 ±1.2 | 4.3 ±0.3 | 24 ±2 | 15.3 ±0.9 | 0.38 ±0.03 | 0.29 ±0.06 |  | 4.0 ±0.55 | 34 ±3.0 |
| **Farming system** |  |  |  |  |  |  |  |  |  |
| Conventional (n=24) | 15 ±1.2 | 3.7 ±0.2 | 21 ±2 | 12.9 ±1.0 | 0.32 ±0.03 | 0.20 ±0.02 |  | 2.5 ±0.40 | 34 ±2.7 |
| Organic (n=30) | 20 ±1.6 | 5.7 ±0.6 | 25 ±2 | 18.8 ±1.2 | 0.45 ±0.05 | 0.39 ±0.07 |  | 4.6 ±0.62 | 40 ±4.1 |
| **ANOVA**(p-values) |  |  |  |  |  |  |  |  |  |
| ***Main Effects*** |  |  |  |  |  |  |  |  |  |
| Year(YR) | **0.0001** | *0.0972* | **0.0005** | **<0.0001** | **0.0022** | NS |  | **0.0076** | **0.0160** |
| Species (SP) | 0.9749 | *0.0585* | NS | **0.0036** | NS | NS |  | NS | *0.0535* |
| Farming system (FS) | **0.0006** | **0.0021** | *0.0538* | **<0.0001** | **0.0356** | **0.0258** |  | **0.0038** | NS |
| ***Interactions*** |  |  |  |  |  |  |  |  |  |
| **YR × SP** | NS | *0.0545* | NS | **0.0044 ^1^** | **0.0394 ^1^** | NS |  | NS | NS |
| **YR × FS** | NS | NS | **0.0335 ^2^** | NS | **0.0269 ^2^** | NS |  | NS | NS |
| **SP × FS** | NS | NS | NS | NS | NS | NS |  | **0.0092 ^3^** | NS |
| **YR × SP × FS** | NS | NS | *0.0781* | **0.0034 ^4^** | *0.0659* | NS |  | NS | NS |
| **^1^**See table S31.1 for Interaction means ± SE; **^2^**See table S31.2 for Interaction means ± SE; **^3^**See table S31.3 for Interaction means ± SE; ^4^See table S31.4 for Interaction means ± SE; | | | | | | | | | |

| ***Table S31.1*.** Interactions means ± SE for the effects of year and species on Zn and Mo content in UK whole-grain flour collected between 2015 and 2016 (results are expressed on a flour dry weight basis) | | | |
| --- | --- | --- | --- |
|  | **Factor 1** | **Factor 2** | |
|  |  | **Species** | |
| **Parameter** | **Year** | spelt | Wheat |
| **Zn** | 2015 | 27 ±2 **A a** | 19 ±1 **B a** |
| mg/kg (DW) | 2016 | 12 ±1 **A b** | 12 ±1 **A b** |
| **Mo** | 2015 | 0.66 ±0.14 **A a** | 0.46 ±0.03 **B a** |
| mg/kg (DW) | 2016 | 0.26 ±0.03 **A b** | 0.32 ±0.04 **A b** |
| For each parameter assessed means labelled with the same capital letter within the same row and same lower-case letter within the same column are not significant different (Tukey’s honestly significant difference test P<0.05) | | | |

| ***Table S31.2*.** Interactions means ± SE for the effects of year and species on Fe and Mo content in UK whole-grain flour collected between 2015 and 2016**.** | | | |
| --- | --- | --- | --- |
|  | **Factor 1** | **Factor 2** | |
|  |  | **Farming system** | |
| **Parameter** | **Year** | Conventional | Organic |
| **Fe** | 2015 | 30 ±2 **A a** | 30 ±1 **A a** |
| mg/kg (DW) | 2016 | 15 ±1 **B b** | 21 ±3 **A b** |
| **Mo** | 2015 | 0.51 ±0.03 **A a** | 0.53 ±0.09 **A a** |
| mg/kg (DW) | 2016 | 0.21 ±0.02 **B b** | 0.38 ±0.04 **A b** |
| For each parameter assessed means labelled with the same capital letter within the same row and same lower-case letter within the same column are not significant different (Tukey’s honestly significant difference test P<0.05) | | | |

| ***Table S31.3*.** Interactions means ± SE for the effects of species and farming systems on Al content in UK whole-grain flour collected between 2015 and 2016 (results are expressed on a flour dry weight basis) | | | |
| --- | --- | --- | --- |
|  | **Factor 1** | **Factor 2** | |
|  |  | **Farming system** | |
| **Parameter** | **Species** | Conventional | Organic |
| **Al** | Spelt | 2.89 ±1.22 **A a** | 3.19 ±0.62 **A b** |
| mg/kg (DW) | Wheat | 2.40 ±0.38 **B a** | 5.72 ±0.91 **A a** |
| For each parameter assessed means labelled with the same capital letter within the same row and same lower-case letter within the same column are not significant different (Tukey’s honestly significant difference test P<0.05) | | | |

| ***Table S31.4*.** Interactions means ± SE for the effects of species, farming system and flour type Zn content in UK whole-grain flour collected between 2015 and 2016 (results are expressed on a flour dry weight basis) | | | | |
| --- | --- | --- | --- | --- |
|  | **Factor 1** | **Factor 2** | **Factor 3** | |
|  |  |  | **Farming system** | |
| **Parameter** | **Year** | **Species** | Conventional | Organic |
| **Zn**  mg/kg (DW) | 2015 | Spelt | 17.3 ±0.0 **B a** | 28.7 ±1.0 **A a** |
|  |  | Wheat | 18.6 ±1.0 **A a** | 19.9 ±1.1 **A b** |
|  | 2016 | Spelt | 10.7 ±1.0 **A b** | 13.7 ±0.8 **A c** |
|  |  | Wheat | 9.1 ±0.6 **B b** | 15.7 ±1.5 **A c** |
| For each parameter assessed means labelled with the same capital letter within the same row and same lower-case letter within the same column are not significant different (Tukey’s honestly significant difference test P<0.05) | | | | |
